# Complexation in Aqueous Solution of a Hydrophobic Polyanion (PSSNa) Bearing Different Charge Densities with a Hydrophilic Polycation (PDADMAC)

**DOI:** 10.3390/polym14122404

**Published:** 2022-06-14

**Authors:** Nouha Jemili, Martin Fauquignon, Etienne Grau, Nicolas Fatin-Rouge, François Dole, Jean-Paul Chapel, Wafa Essafi, Christophe Schatz

**Affiliations:** 1 Laboratoire Matériaux, Traitement et Analyse, Institut National de Recherche et d’Analyse Physico-Chimique, Pôle Technologique de Sidi Thabet, 2020 Sidi Thabet, Tunisia; nouhajem@hotmail.com; 2 Université de Tunis El Manar, Faculté des Sciences de Tunis, 2092 Tunis, Tunisia; 3 Laboratoire de Chimie des Polymères Organiques (LCPO), Université de Bordeaux, CNRS, Bordeaux INP, UMR 5629, 33600 Pessac, France; martin.fauquignon@enscbp.fr (M.F.); etienne.grau@enscbp.fr (E.G.); 4 Institut de Chimie des Milieux et des Matériaux de Poitiers, Université de Poitiers, 86073 Poitiers, France; nicolas.fatin-rouge@univ-poitiers.fr; 5 Centre de Recherche Paul Pascal (CRPP), UMR CNRS 5031, Université de Bordeaux, 33600 Pessac, France; dole@crpp-bordeaux.cnrs.fr

**Keywords:** hydrophobic/hydrophilic polyelectrolytes, pearl necklace conformation, polyelectrolyte complexes, complex particles

## Abstract

In this work the electrostatic complexation of two strong polyelectrolytes (PEs) was studied, the hydrophilic and positively charged poly (diallyldimethylammonium chloride) (PDADMAC) and the hydrophobic and negatively charged poly (styrene-*co*-sodium styrene sulfonate) (P(St-co-SSNa)), which was prepared at different sulfonation rates. The latter is known to adopt a pearl necklace conformation in solution for intermediate sulfonation rates, suggesting that a fraction of the P(St-co-SSNa) charges might be trapped in these hydrophobic domains; thus making them unavailable for complexation. The set of complementary techniques (DLS, zetametry, ITC, binding experiment with a cationic and metachromatic dye) used in this work highlighted that this was not the case and that all anionic charges of P(St-co-SSNa) were in fact available for complexation either with the polycationic PDADMAC or the monocationic *o*-toluidine blue dye. Only minor differences were observed between these techniques, consistently showing a complexation stoichiometry close to 1:1 at the charge equivalence for the different P(St-co-SSNa) compositions. A key result emphasizing that (i) the strength of the electrostatic interaction overcomes the hydrophobic effect responsible for pearl formation, and (ii) the efficiency of complexation does not depend significantly on differences in charge density between PDADMAC and P(St-co-SSNa), highlighting that PE chains can undergo conformational rearrangements favoring the juxtaposition of segments of opposite charge. Finally, these data have shown that the formation of colloidal PECs, such as PDADMAC and P(St-co-SSNa), occurs in two distinct steps with the formation of small primary complex particles (<50 nm) by pairing of opposite charges (exothermic step) followed by their aggregation within finite-size clusters (endothermic step). This observation is in agreement with the previously described mechanism of PEC particle formation from strongly interacting systems containing a hydrophobic PE.

## 1. Introduction

Polyelectrolyte complexes (PECs) are formed through predominant cooperative electrostatic interactions between oppositely charged polyelectrolytes (PEs) upon mixing their aqueous solutions together [1,2,3,4,5]. Many industrial applications of PECs exist, such as papermaking [6], food industry [7], wastewater treatment [8], pharmaceutical industry, and biomedicine [9]. The driving force of PEC formation is mainly the gain in entropy due to the release of the low-molecular counterions (and bound water molecules). However, other interactions, such as hydrogen bonding or hydrophobic ones, may play an additional part [2]. Two types of complexes can be distinguished on the basis of their physical nature, i.e., solid or liquid, the latter belonging to the broader family of coacervates [10]. Intermediate physical states can be observed as gel-like states, evidencing the existence of a continuum of morphology depending on the interaction strength between the two oppositely charged PEs or the complexation conditions used (temperature, ionic strength, solvent) [11]. When the PE mixture is not at charge stoichiometry, complexation generally gives rise to colloidal particles where the PE in excess forms an electrosterically stabilizing shell around the neutral complexed cores [12]. Except for the case of soluble complexes discovered by Kabanov and Zezin, which are formed under particular conditions [13], colloidal PEC particles can be obtained from almost any pairs of PEs as long as a PE is in excess and the concentration is not too high (<1%) [2,14]. The major component, which is bound on the surface, can stabilize particles up to the 1:1 mixing ratio, where flocculation or coalescence occurs, depending on whether particles are solid- or liquid-like complexes, respectively. However, in many cases, premature particle aggregation can be observed, i.e., at mixing ratios different from 1:1. This issue has been reviewed from a theoretical perspective, describing aggregation as occurring when short-range attractions (van der Waals, hydrophobic) overcome long-range electrostatic repulsion between like-charge complexes [15]. 

**Figure 1:**
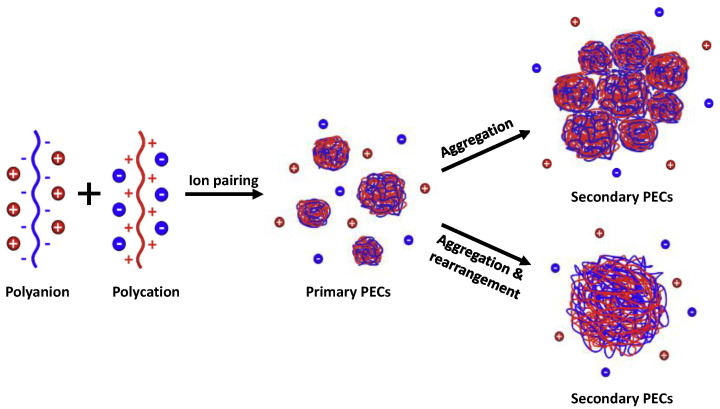
Two-step mechanism of PEC particle formation. The morphology of the secondary complexes depends on the hydrophobicity of the PEs, with highly hydrophobic PEs favoring a raspberry-shaped morphology and hydrophilic PEs leading to a more homogeneous structure (adapted from [16]).

For solid-like complexes, the formation of PEC particles is generally described by a two-step mechanism, which consists in the formation of primary PEC particles, followed by growth of secondary PECs from the aggregation of the primary particles (Figure 1) [16,17]. When PEs are hydrophilic, the secondary PECs have a rather homogeneous structure, meaning that primary complexes can rearrange and fuse into quite homogeneous particle structure. For hydrophobic PEs, the situation is different as small primary complex particles are too compact to rearrange; consequently, they aggregate to larger raspberry-like particles [18]. For example, Gummel et al. have shown that complexes based on hydrophobic polystyrene sulfonate and lysozymes form dense globules of finite size (~10 nm) arranged into fractal aggregates that are resistant to dilution [19,20]. 

**Figure 2:**
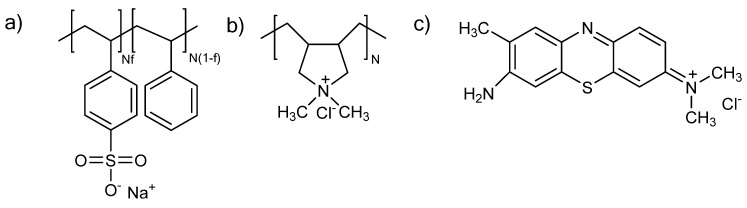
Molecular structure of (**a**) P(St-co-SSNa), (**b**) PDADMAC, (**c**) *o*-toluidine blue. N is the degree of polymerization and *f* represents the chemical fraction of sulfonated units in P(St-co-SSNa).

In the present study, we investigate the complexation between a hydrophilic cationic polyelectrolyte, the poly (diallyldimethylammonium chloride) (PDADMAC) and a hydrophobic polyanion, the poly (styrene-*co*-sodium styrene sulfonate) with varying degrees of sulfonation (Figure 2). Both PEs are strong; their electrostatic charges are quenched and almost pH independent. PDADMAC and PSSNa have both been considered as model polyelectrolytes [21,22]. The complexation of PDADMAC and PSSNa in dilute conditions has been extensively investigated by the group of Dautzenberg in the 1990s by static light scattering giving access to the radius of gyration, the molecular weight, the polydispersity and the structural density of PEC particles [23,24]. In pure water, PEC particles obtained from PDADMAC of 250,000 g/mol and PSSNa of 66,000 g/mol have a high level of aggregation with more than 1000 chains per particle as well as a high polydispersity [12]. Electron microscopic studies indicated that PEC particles have a spherical shape and a compact structure and confirmed the high size polydispersity found by light scattering [14]. At that time, the substructure of PEC particles could not be revealed. 

The charge density of PSSNa can be modified through partial sulfonation of the parent polystyrene chain, leading to the poly (styrene-*co*-sodium styrene sulfonate), abbreviated as P(St-co-SSNa). The physicochemical behavior of this hydrophobic PE is clearly different from the fully charged PSSNa, especially regarding the chain conformation. At first, it was predicted theoretically by Dobrynin and Rubinstein that hydrophobic PEs adopt in water a pearl necklace conformation where dense beads are connected by narrow strings [25,26]. The pearl size results from the balance between the electrostatic energy and the interfacial energy while the distance between pearls results from the balance between the repulsion between adjacent pearls and the surface energy of the string [25,26]. As the hydrophobic polyelectrolyte becomes more charged, the size of the pearls decreases and their number increases, by analogy with the Rayleigh instability of a charged droplet [27]. Experimentally, the average form factor of P(St-co-SSNa) with chemical charge fractions (*f*) of *f* = 0.36 and *f* = 0.64 was measured by small-angle neutron scattering combined with the zero average contrast method, highlighting the pearl necklace conformation and also a decrease in the pearl size as *f* increases [28]. Light scattering and pyrene fluorescence measurements also suggested the formation of hydrophobic nano-domains dispersed along the polymer chain [29,30]. Importantly, Essafi et al. showed by osmometry that the effective charge fraction (*f_eff_*) of P(St-*co*-SSNa) (0.35 < *f <* 1) was much lower than predicted by the Manning–Oosawa condensation theory [31,32], except for *f* = 1 [33]. The reduction of the effective charge was attributed to the low local dielectric constant in the pearls, thereby favouring ion condensation. In contrast, no further reduction of *f_eff_* was found for a random copolymer of acrylamide and sodium-2-acrylamide-2-methyl propane sulfonate, due to its highly hydrophilic behaviour.

Investigations of P(St-co-SSNa) on adsorbed surfaces align with the pearl necklace conformation as the size of the pearl was quantitatively measured by *in situ* ellipsometry [27]. Moreover, it was shown by AFM imaging that P(St-co-SSNa) chains of low charge density (*f* < 0.5) are collapsed in spherical globules, while highly charged chains have a more extended conformation [34]. It appears thus at the limit of solubility in water, the less charged P(St-co-SSNa) form spherical globules, like oil in water. As the charge density increases, the system becomes unstable and the spherical globules split into smaller ones.

In the context of polyelectrolyte complexation, the question of the charge accessibility within the pearls arises. Are they accessible in the same way as those located on the strings? Are the counter ions in the pearls more difficult to remove, i.e., is there an additional energy cost to provide? To address these and other questions, we first studied the chain conformation and solution dynamics of P(St-co-SSNa) with varying sulfonation rates by AFM and dynamic light scattering (DLS), respectively. Then, we investigated the complexation of P(St-co-SSNa) with PDADMAC by DLS, zetametry, electron microscopy, and isothermal titration calorimetry (ITC) in order to examine the size, charge, morphology of PEC particles and thermodynamics of complexation. The complexation of P(St-co-SSNa) with a monocationic dye, the toluidine blue, was also studied by spectrophotometric titration for comparison with the PDADMAC. Special attention was paid to possible differences in complexation stoichiometry by forming PEC particles at different molar charge ratios (Z = [+]/[−]).

## 2. Materials and Methods

**Polymers.** Poly (diallyl dimethylammonium chloride) (PDADMAC), M_w_ ≤ 100,000 g mol^−1^ (M_w_ as the weight averaged molecular weight), N = 619 (N is the polymerization index) was purchased from Sigma Aldrich (Saint Louis, MO, USA) as a 35 wt. % solution in H_2_O. Before use, the PDADMAC solution was dialyzed against Milli-Q water using regenerated cellulose membranes (MWCO 6–8 kDa Spectra/Por) until the conductivity of the dialysis bath was constant. Then, the dialyzed solution was concentrated and freeze dried. P(St-co-SSNa) with different degrees of sulfonation *f* (45%, 64%, 83% and 100%) were used as hydrophobic polyanions. They were obtained by post sulfonation of a parent polystyrene with M_w_ = 280,000 g mol^−1^, N = 2692, purchased from Sigma Aldrich (Reference 182427). The sulfonation method was derived from a previous procedure developed by Essafi et al. [35], based on Makowski’s method [36], allowing a partial sulfonation of polystyrene with a good control of the composition [37]. After the sulfonation step, all P(St-co-SSNa) copolymers were purified by dialysis and freeze dried, as described above. The water content of each polymer was determined by thermogravimetric analyses (TGA 5500, TA instruments, New Castle, DE, USA). 

The degrees of sulfonation of P(St-co-SSNa) were obtained by two methods: (i) elemental analysis of carbon and sodium (Flash EA 1112, Thermo Finnigan, Waltham, MA, USA) (Table S1) and (ii) ^1^H NMR analyses (Ultrashield Plus 500, Bruker, Billerica, MA, USA) of the copolymers in deuterated DMSO or D_2_O (Figure S1) [37]. The two methods gave similar sulfonation degrees. The main characteristics of PDADMAC and P(St-co-SSNa) are summarized in Table 1. 

**Chain conformations ofP(St-co-SSNa)** were determined using VMD ((1.94a51, University of Illinois, Urbana-Champaign, IL, USA, https://www.ks.uiuc.edu/Research/vmd/, accessed on 13 May 2022) and AMS (AMS2021, SCM Software for Chemistry & Materials BV, Amsterdam, The Netherlands, https://www.scm.com/, accessed on 13 May 2022) softwares. In a first step, a polymer chain of 100 randomly generated units was built. Then, the chain was relaxed using the universal force field (UFF). The radial distribution functions between sulfur atoms were generated using VMD (Figure S2). For each sulfur atom, the distances to the nearest sulfur atom were calculated and averaged (Table 1).

**Preparation of PE solutions and complex formation.** Polyelectrolyte solutions were obtained by dissolving the polymer powder in Milli-Q water (Resistivity = 18.2 MΩ cm^−1^) at a concentration of 1.5 mM in charged units by taking into account the water content and the sulfonate content. PE solutions were prepared at room temperature and kept under constant stirring for at least 15 h and filtered through 0.2 µm cellulose acetate filters. The formation of PEC particles was performed by rapid addition of the PDADMAC solution into the P(St-co-SSNa) solution under stirring for Z < 1 and by reversing the order addition for Z > 1 so that the component in default was always added to the one in excess in order to minimize the PEC aggregation upon crossing the point of charge equivalency (Z = 1). Different values of the molar charge ratios Z = [+]/[−] were targeted. The total PE concentration was always equal to 1.5 mM in charged units. The suspensions were then promptly used for DLS and zeta potential measurements to avoid any significant sedimentation. 

**Transmission electron microscopy (TEM).** Transmission electron microscopy (TEM) experiments were performed using a Hitachi H600 (Hitachi, Tokyo, Japan) microscope operating at an acceleration voltage of 75 kV. A droplet of PEC dispersion was deposited on conventional carbon-coated copper grids and TEM images were taken after complete drying of the sample at room temperature.

**Dynamic light scattering (DLS) and zetametry.P(St-co-SSNa) solutions.** Dynamic light scattering analyses of aqueous solutions of P(St-co-SSNa) were performed using a compact ALV-CGS3 goniometer with an ALV/LSE-5004 light scattering electronics and an ALV-7004 multi tau digital correlator with pseudo-cross correlation detection (ALV-Laser Vertriebsgesellschaft mbH, Langen, Germany). The light source was a 22 mW He-Ne laser operating at λ = 632.8 nm. The analysis was carried out at 25 °C at a single detection angle of 90°. A total of 10 measurements of 30 s were performed, except for PSS100% where 60 measurements of 120 s were performed due to the low scattering intensity. Data were evaluated by fitting the normalized time autocorrelation function (ACF) of the scattered light intensity, $g_{\left(2\right)}\left(t\right)$ which is related to the normalized electric field ACF, $g_{\left(1\right)}\left(t\right)$ through the Siegert relation: $g_{\left(2\right)}\left(t\right)=1+\beta \left|g_{\left(1\right)}\left(t\right)\right|$^2^ where *β* is an instrumental constant. The data were fitted with the constrained regularization method (CONTIN), which provides the distribution of relaxation times, *A(τ)*, as the inverse Laplace transform of the electric field ACF: $g_{\left(1\right)}\left(t\right)=\int_{0}^{\infty }A\left(\tau \right)\exp\left(-t/\tau \right)d\tau$. The distribution of relaxation times was converted to an intensity-weighted size distribution by applying the Stokes–Einstein equation. The P(St-co-SSNa) solutions were prepared at a concentration of 6 mM in charged units (=1.2 g/L for PSS100%, 1.4 g/L for PSS83%, 1.6 g/L for PS64%, 2.0 g/L for PSS45%) and filtered on 0.22 µm cellulose acetate filters just before analysis. 

**PEC particles.** Dynamic light scattering and zeta potential measurements of PEC particles at various molar charge ratios (Z) were carried out using a NanoZS Zetasizer (Malvern Instruments, UK), operating with a 4 mW He−Ne laser (λ_0_ = 632.8 nm) and a detection angle of 173°. The determination of zeta potential (ZP) uses a combination of laser Doppler velocimetry and phase analysis light scattering (PALS). The prepared PEC dispersions were introduced in folded capillary cells (DTS1070) to perform successively DLS and ZP measurements at 25 °C. The particle size distributions were obtained by applying the non-negative least squares (NNLS) fitting model. When the distributions were monomodal, the particle Z-average diameter was derived by applying the cumulant model. For the determination of ZP values, the Smoluchowski approximation was applied. 

**Atomic force microscopy (AFM).** P(St-co-SSNa) chains deposited on mica surfaces were imaged in air at room temperature using a Dimension ICON AFM (Bruker, Billerica, USA). Experiments were made in PeakForce mode to precisely control the force acting on the chains (setpoint) using high-resolution PeakForce HIRS-FA probes having a tip radius of ~1–2 nm (Bruker, Billerica, MA, USA) at a scanning rate of 0.4 Hz. Mica substrates were freshly cleaved prior to any experiment to expose a genuine and molecular smooth surface. It must be noted that the mica and P(St-co-SSNa) chains are both negatively charged in aqueous solution, which prevents adsorption of the polyanions. Mica substrates were then immersed for 10 s into a 2 mM MgCl_2_ solution, since divalent cations, such as Mg^2+^, are known to electrostatically bridge with polyanions, such as PSSNa or DNA [34,39,40]. In total, 200 µL of aqueous P(St-co-SSNa) solutions prepared at 1 mM were subsequently cast on the pre-treated mica surfaces for a few minutes to promote chain adsorption, rinsed with DI water, and dried under a clean nitrogen stream prior to AFM imaging.

**Isothermal titration calorimetry (ITC).** A Nano ITC calorimeter from TA Instruments (New Castle, DE, USA), with a sample cell and syringe volume of 950 µL and 250 µL, respectively, was used to determine the heat transfer during the complexation of the oppositely charged PEs. The PDADMAC solution (c = 6 mM in monomers) was introduced in the syringe (titrant) and injected in the working cell containing the P(St-co-SSNa) titrated solution (c = 1 mM in monomers) under constant stirring (250 rpm). All solutions were degassed before each experiment, including the water filled in the reference cell [41,42]. After a preliminary injection of 2 μL of the titrating PE solution, 24 successive injections of 10 μL with a 300 s time interval between each injection, were performed. The first point was not included in the analysis due to the diffusion of the solution into the needle during the equilibration time. All experiments were conducted at 25 °C. The heat flow corresponding to each injection was recorded as a function of time and integrated. The heat of dilution from the PDADMAC addition into deionized water was measured and subtracted from the raw data (Figure S4). The experimental data were fitted with a model of two independent binding sites, allowing the complexation of PDADMAC with P(St-co-SSNa) to be described as a two-step process [43,44]. The binding constant (K), the reaction stoichiometry (n), and the enthalpy change per mole of injectant (ΔH) were determined for each process with the NanoAnalyze software (version 3.11.0, TA Instruments, New Castle, DE, USA). The free energy change (ΔG) and entropy change (ΔS) were derived from the fitted parameter values using thermodynamic equations, ΔG = − RT ln K and ΔG = ΔH − TΔS, where R is the gas constant and T is the absolute temperature.

**Spectrophotometric titration.** P(St-co-SSNa) solutions were spectrophotometrically titrated with *o*-toluidine blue (Sigma Aldrich, 84% purity, used as received). A total of 1.35 mL of the dye solution prepared at 6.8.10^−5^ M was rapidly added to 0.15 mL of P(St-co-SSNa) solutions varying in concentration, from 5.10^−5^ M to 1.3.10^−3^ M (in charged residues). Solutions were incubated at 25 °C under stirring for one hour. Following this, triplicates were pipetted into a 96 well plate (200 µL/well) and analyzed with a microplate reader (SpectraMax M2e, Molecular devices) in the visible range (400–800 nm). Solutions were also centrifuged (20 min at 20,000× *g*) afterwards to better evidence the formation of precipitate under certain conditions.

## 3. Results

### 3.1. Solution Behavior and Conformation of P(St-co-SSNa)

Before investigating the formation of P(St-co-SSNa) complexes with PDADMAC, the solution behavior and chain conformation of P(St-co-SSNa) were studied by DLS and AFM, respectively. All P(St-co-SSNa) solutions were prepared at a concentration of 6 mM in charged units, which corresponds to a mass concentration of 1.2 g/L for PSS100% (Mw = 550,000 g/mol). The overlap concentration of the PSS100% was estimated at 0.05–2 g/L according to the literature [45]. Therefore, the solutions were analyzed at the onset of the semi-dilute regime, but far from the entangled regime (c_e_~40 g/L for PSS100%) [45]. 

**Figure 3:**
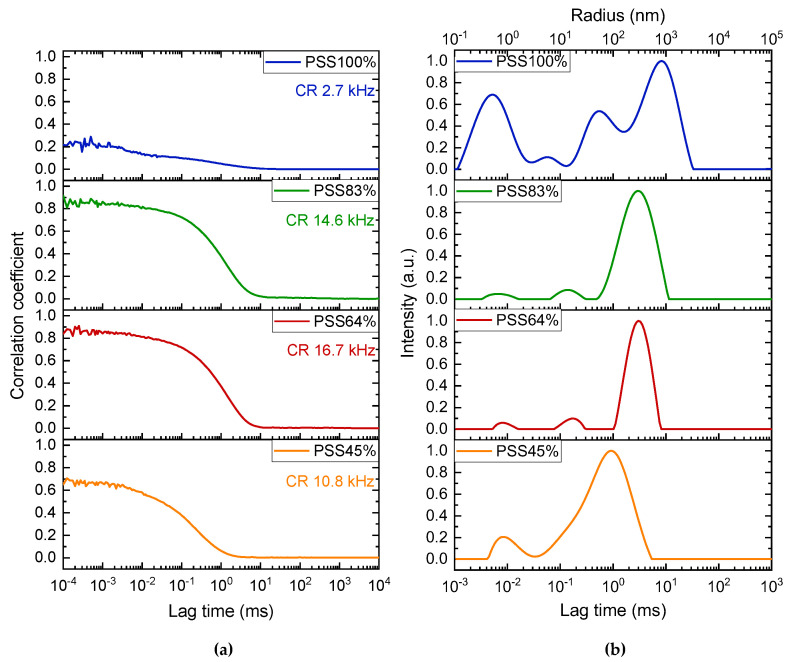
DLS analysis of aqueous solutions of P(St-co-SSNa) varying in the degree of sulfonation. (**a**) Normalized intensity autocorrelation functions. The scattering intensity of P(St-co-SSNa) solutions is given as the count rate (CR) determined at 90° detection angle and normalized by the polymer mass concentration (g/L). (**b**) Intensity-average relaxation time distributions. The corresponding unweighted radii are shown on the top axis. All PE solutions were prepared at a concentration of 6 mM in charged units.

**Figure 4:**
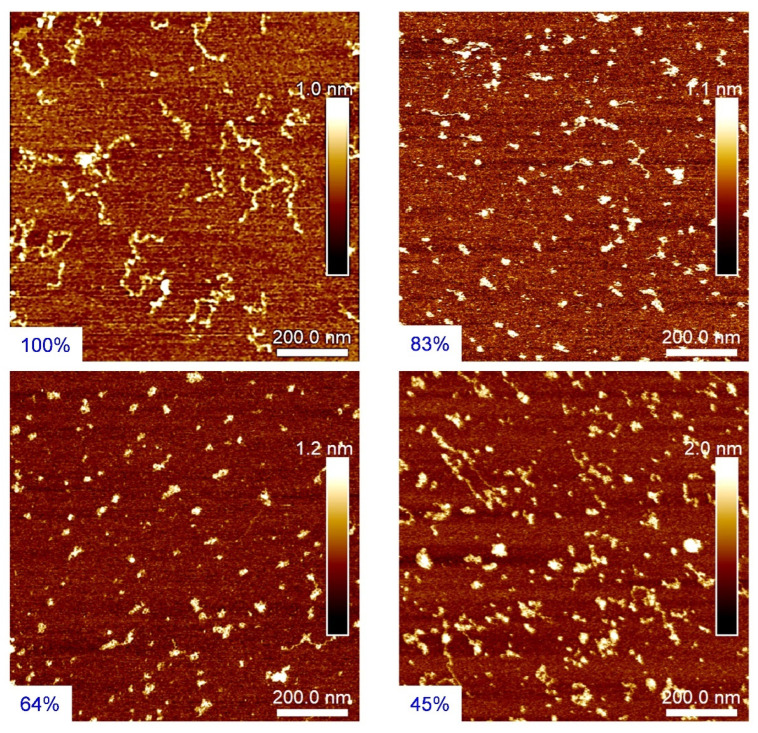
AFM images of PSS100%, PSS83%, PSS64%, and PSS45% chains on molecularly smooth MgCl_2_-treated mica surfaces.

Starting with the DLS analysis, the P(St-co-SSNa) with a degree of sulfonation of 100 % (PSS100%) exhibits a typical polyelectrolyte behavior in salt-free solution [46], which is characterized by: (i) a low scattering intensity in relation with the low compressibility of the charged polymer (Figure 3a), (ii) a fast relaxation mode (~10 µs) that can be attributed to the coupled relaxation of polymer charges and their counter-ions (Figure 3b), (iii) a slow relaxation mode (~10 ms) resulting from transient polymer associations due to the interaction of charge-ion dipoles [47], leading to ‘electrostatic aggregates’ (Figure 3b), and (iv) medium modes (Figure 3b) that can be ascribed to the polydispersity of the polymers [48] (see Figure 4), as generally observed for PSSNa obtained by post sulfonation of PS [34,49,50]. The low intercept on the correlogram (0.2) also reflects the poor coherence of the scattering fluctuations, probably due to the low scattering intensity and the high dispersity of relaxation times spanning five decades (Figure 3a). Partially sulfonated PSS (PSS83%, PSS64%, PSS45%) showed a somewhat different behaviour: (i) the scattering level is higher due to a higher compressibility of these PSSNa since their effective charge in aqueous media decreases as *f* decreases and probably also due to the pearl necklace conformation [30]; the lower count rate observed with the more hydrophobic PSS45% could be due to the removal of some aggregates upon filtration. The increase in scattering intensity with the decrease of the sulfonation rate was also confirmed by measurements of the refractive index increment (dn/dc) of P(St-co-SSNa) (Table S2); (ii) the fast mode is of lower intensity due to the decrease in polymer charge density; (iii) medium modes can still be observed; and (iv) the slow mode is detected at shorter times and it is also less broad than for PSS100%; it can be attributed to the formation of denser aggregates through hydrophobic interaction rather than ion-dipole interaction. The aggregate size was determined by application of the Stokes–Einstein equation; it decreases from 250 nm (PSS64% and PSS83%) to 100 nm (PSS45%).

Figure 4 shows AFM images of P(St-co-SSNa) chains deposited on molecularly smooth MgCl_2_-treated mica surfaces. The electrostatic adsorption of negatively charged chains onto the cationic mica surface is here driven by the release of counterions. The AFM images showed essentially individual chains and none of the large aggregates observed by DLS. The latter diffuse much more slowly than the individual chains, so they likely did not have time to adsorb during the deposition step on the mica surfaces. For the highest charge density (PSS100%), the polyelectrolyte chains appeared to lie flat on the surface in a rather extended conformation with a height of about 0.7 nm, in agreement with the radius of a PSSNa chain and the previous work of Gromer et al. [34]. We could further see that the adsorbed chains of the fully charged PSSNa appeared quite polydisperse on the mica surface with the presence of very small chains (and a few branched structures). An observation consistent with that of Gromer et al. [34] and Baigl et al. [37] suggesting that some chain scissions (and/or crosslinking) may have occurred during the sulfonation step. For the two intermediate charge densities (PSS64% and PSS83%), the chains were less extended and presented slightly higher heights (see scale bars). Finally, for the lowest charge density (PSS45%), the chains appeared more folded with heights often greater than 1.5 nm and the presence of small aggregates, which certainly underlines the marked hydrophobic nature of these low charge density polyanions chains. However, not all chains were aggregated, suggesting that aggregates have a weakly cohesive structure and that their interaction with the cationic charges (Mg^2+^) of the mica surface could be sufficient to partially unwind them. A feature that will also be observed in solution upon complexation of low charge density P(St-co-SSNa) with the hydrophilic and cationic PDADMAC.

### 3.2. Observations of P(St-co-SSNa)-PDADMAC Complexes

**Figure 5:**
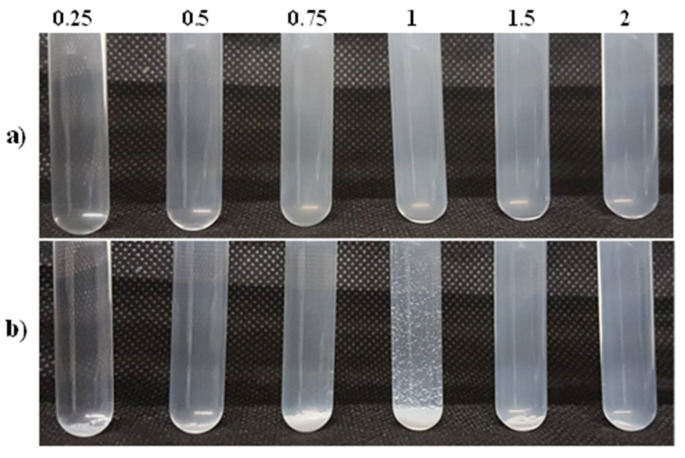
Optical aspect of the PEC dispersions formed by rapid addition of PDADMAC into PSS100% at various molar charge ratios Z(+/−), between 0.25 and 2. (**a**) immediately after mixing; (**b**) after five days.

In this section, PECs were prepared at different molar charge ratios Z (+/−) by rapid addition of the PDADMAC solution to the PSS100% solution. The qualitative aspect of the PEC dispersions obtained just after mixing is shown in Figure 5a. An optically transparent solution was obtained at the lowest charge ratio (Z = 0.25). At Z = 0.5 and Z = 0.75, the dispersions were slightly turbid due to the formation of more colloidal complexes. At Z = 1, which corresponds to the charge equivalency, the highest turbidity was observed. A further increase of the molar charge ratio led to less turbid dispersions. Five days after preparation of the complexes, some of the PEC particles precipitated for 0.75 ≤ Z ≤ 1.5 with a maximum of precipitation at Z = 1, in good agreement with the achievement of the complete charge neutralisation in the system, as previously reported for the PDADMAC-PSSNa pair [17] (Figure 5b). 

**Figure 6:**
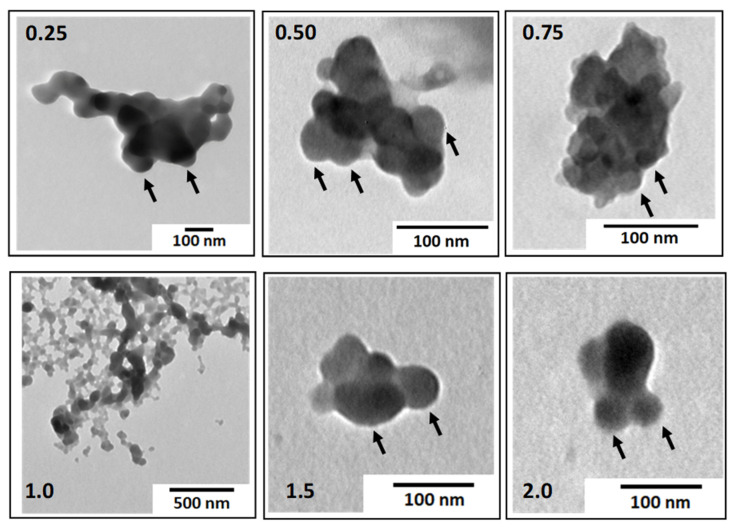
TEM images showing the morphology of PEC particles obtained by complexing PDADMAC with PSS100% at various molar charge ratios Z(+/−) comprised between 0.25 and 2. The arrows highlight the substructure of PEC particles where primary complexes can be observed.

Then, the PEC morphology at different charge ratios was examined by TEM (Figure 6). It can be observed at first sight that PEC particles have irregular shapes with sizes in the range of 100 to 200 nm for Z values different from 1. However, a closer look at the images, for example at Z = 0.50, clearly shows that the complex particles are made up of smaller particles interacting with each other. These particles, whose size was typically comprised between 20 and 50 nm, correspond to the primary complexes previously described. Primary complexes should be charged at Z < 1 or Z > 1 because of the excess PE that is partially complexed on their surface. The clustering of these small particles into larger structures of finite size may result from the interplay of short-range attractive interactions (van der Waals), which allow for the minimization of the surface energy of the particles upon aggregation [51] and long-range repulsive electrostatic interactions [15,52]. The growth of secondary complexes is limited by the increasing electrostatic energy of the clusters [51]. The fact that the primary complexes cannot merge and rearrange (Figure 1) emphasizes their dense and cohesive structure due to their hydrophobicity. In the case of more hydrophilic PE systems, this type of hierarchical structure does not exist, i.e., the primary complexes, if any, can fuse and rearrange [18,53].

### 3.3. Characterization of P(St-co-SSNa)-PDADMAC Complexes by DLS and Zetametry

**Figure 7:**
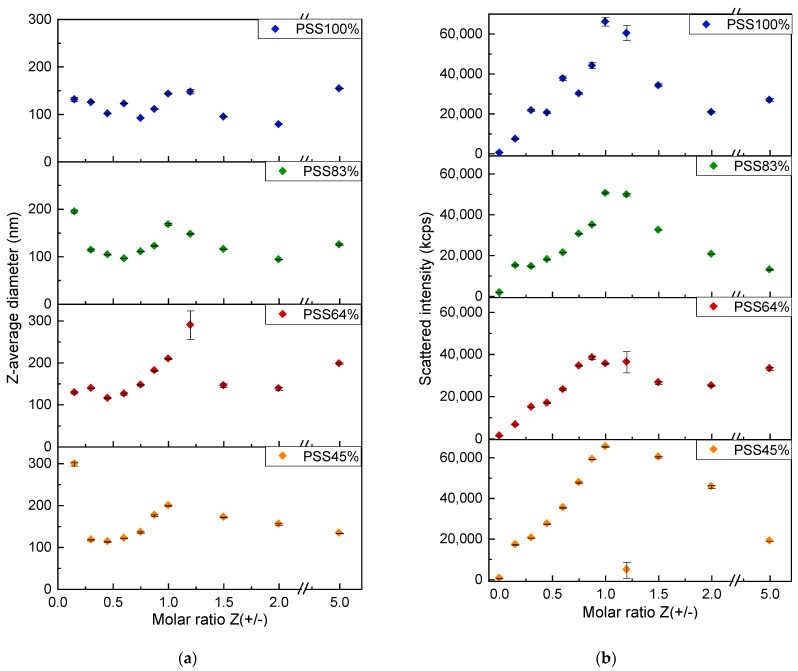
DLS analysis of PDADMAC/P(St-co-SSNa) complexes in solution. The Z-average diameter of the particles (starting at Z = 0.15) (**a**) and the scattering intensity of the dispersion (starting at Z = 0) (**b**) are plotted for the four P(St-co-SSNa) compositions as function of the molar charge ratio Z(+/−). The total PE concentration was 1.5 mM in charged units.

In order to probe the colloidal state of the complexes and to determine the stoichiometry of the system as precisely as possible, the complexes prepared at different Z(+/−) by rapid addition of PDADMAC to P(St-co-SSNa) for Z < 1 or P(St-co-SSNa) to PDADMAC for Z > 1, were analyzed by DLS within a few minutes after their preparation. Indeed, after a certain time, a fraction of PEC particles sedimented, especially at Z between 0.7 and 1.5. The formation of colloidal particles was detected by DLS even for very low charge ratios, which totally excludes the formation of soluble complexes in the sense defined by Kabanov and Zezin [13]. The DLS analysis showed that the size distributions of complexes were essentially monomodal (Figure S3), which allowed for the application of the cumulant method to roughly estimate the Z-average diameters of the particles. PEC particles have diameters between 100 and 300 nm regardless of the sulfonation rate of the P(St-co-SSNa) (Figure 7a). For a given P(St-co-SSNa), the particle sizes varied slightly with Z, as found elsewhere for the PSS/PDADMAC system [12]. However, some aggregation could be detected when approaching Z = 1, as the amount of excess PE was too low to well stabilize the complexes. The aggregation was more visible for P(St-co-SSNa) than for PSS100%. The fact that PEC particles have a finite size confirms the formation mechanism of primary complex clusters, as seen in TEM (Figure 6). Interestingly, one could also detect by DLS a population of low intensity, around 50 nm, even for relatively high charge ratios (Figure S3). This population could correspond to a fraction of unclustered primary complexes. 

It is of importance to note that the scattering intensity increased linearly between Z = 0 and Z = 1 for all P(St-co-SSNa). Assuming that the optical contrast of the complexes was constant, the increase in intensity must be due either to an increase in the number of particles or to an increase in their size. The latter being almost constant for Z < 0.8 (see, for example, the data of PSS100%, Figure 7a), the particle number must then increase. This indirectly reveals that the strong interaction between both PEs leads to a complexation far from equilibrium generating inhomogeneous dispersions of complexes due to a reaction time faster than the mixing time [54,55]. Indeed, equilibrium complexation or homogeneous mixing conditions would not have left almost any free PDADMAC chains in the solution even at low Z as a single PDADMAC chain can interact with several P(St-co-SSNa) chains. For instance, if one assumes that in the early stage of complexation, one PDADMAC chain can interact with five P(St-co-SSNa) chains, there should be no free P(St-co-SSNa) in solution above Z = N_PDADMAC_/(5 *f* N_P(St-co-SSNa)_) ~ 0.05 (with *f* = 1). In other words, most of the complexes must nucleate at low Z under equilibrium or homogeneous mixing conditions and only their growth should be observed at higher Z. Since particle growth was not actually observed, it must be assumed that the complexation was heterogeneous, i.e., both complexed and free P(St-co-SSNa) chains were present in the dispersion at low Z. Then, by adding more PDADMAC, free P(St-co-SSNa) chains can nucleate new particles; thus contributing to the increase of the scattered light. In practice, it is difficult to achieve the complexation in homogeneous conditions since the complexation reaction is an extremely fast process (<5 µs, i.e., nearly the diffusion-collision time of the PE chain) [56]. The use of microfluidic mixers has been proposed to have mixing times lower than the reaction time, and thus achieve homogeneous complexation conditions [57]. 

**Figure 8:**
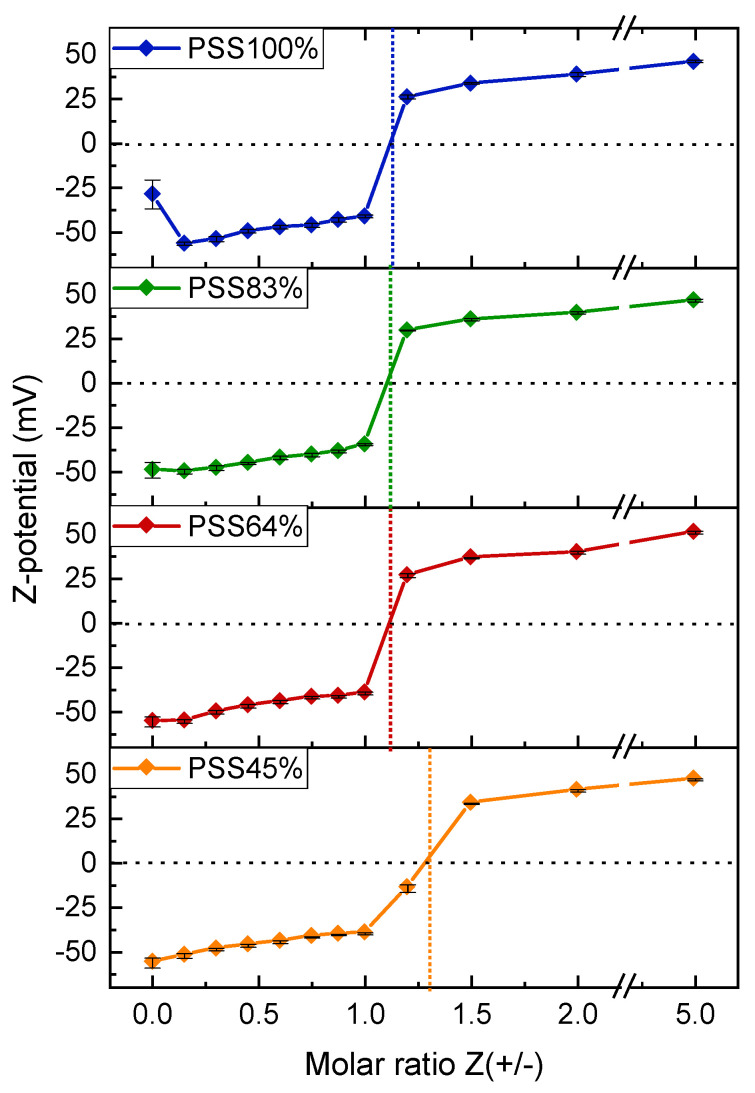
Zeta potential measurements of PDADMAC/P(St-co-SSNa) complexes in solution as function of the molar charge ratio Z(+/−) for the four P(St-co-SSNa) compositions. Zetametry was performed on same samples as those used in Figure 7. The total PE concentration was 1.5 mM in charged units. The point of zero charge is indicated by the dotted lines Solid lines are only to guide eyes.

Finally, the size and scattered intensity variations at Z > 1 were symmetrical to those obtained at Z < 1 (Figure 7). This shows that the complexation mechanism in the presence of excess PDADMAC is similar to that observed with excess P(St-co-SSNa), at least in the case of rapid mixing of the PEs. For Z > 1, primary complexes must be slightly positively charged due to PDADMAC in excess and the same must hold for secondary complexes. The neutrality in strong PEC systems is characterized by a complete charge pairing leading to the precipitation of complexes. This is equivalent to coacervation for weak complexes. Here, precipitation was only observed within a few minutes for PSS45% at Z = 1.2, as evidenced by the drop in scattering intensity (Figure 7b) and by the absence of particles of colloidal sizes detected by DLS (Figure S3). For the other P(St-co-SSNa), a scattered intensity maximum was detected at Z = 1.2 without any precipitation as the particle sizes did not exceed 300 nm (Figure 7a). This emphasizes that the complexes remain relatively well stabilized as long as there is a slight excess of one of the PE (P(St-co-SSNa for Z < 1 or PDADMAC for Z > 1). Therefore, it can be concluded from DLS analysis, that the effective charge stoichiometry of the system must be close to Z = 1.2 with minimal differences among the various P(St-co-SSNa). This suggests that neither the pearl necklace conformation of P(St-co-SSNa) nor the difference in charge density between the two PEs (Table 1) impaired the full complexation of sulfonate groups by the cationic charges of the PDADMAC. This was also confirmed by zeta potential (ZP) measurements, where the charge neutrality corresponding to zero potential was estimated at Z = 1.1 for PSS100%, PSS83% and PSS64% and Z = 1.2 for PSS45% (Figure 8). However, the out-of-equilibrium conditions of the complexation or inhomogeneity of mixing probably explain why the complete charge neutralization was obtained at Z slightly different from 1. The ZP measurements also highlighted an abrupt reversal of the charge of the complexes around the point of zero charge (pzc), which confirms that PEC particles remained highly charged and rather stable even in the presence of a small excess of PE [10] (Figure 8). Furthermore, there was no significant difference in the absolute values of ZP as a function of the sulfonation rate, suggesting that the complexation mechanism and stoichiometry were similar for all P(St-co-SSNa). The ZP value obtained at Z = 0, where only PSS100% chains are present in the solution (Figure 8) is questionable due to the high variability in the values obtained in the measurements (data not shown). First, the Henry’s equation used to determine the ZP is not adapted to the case of polyelectrolyte chains: (i) the ZP is a potential associated with an interfacial charge density and polyelectrolytes do not have an interface, and (ii) no surrounding fluid can flow through the particles, but it can flow through PE coils [58,59]. Second, even though the electrophoretic mobility of PEs can be determined by phase analysis light scattering (PALS), the poor scattering behavior of PSS100% certainly resulted in a poor quality of the zeta potential measured. More consistent zeta potential values were obtained for other P(St-co-SSNa) at Z = 0, probably due to their initial collapsed state.

### 3.4. Characterization of P(St-co-SSNa)-PDADMAC Complexation by ITC

**Figure 9:**
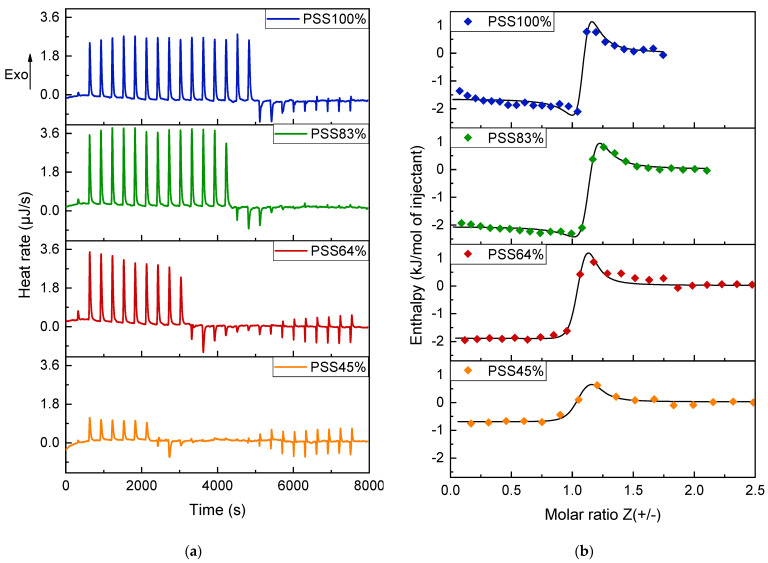
ITC raw data and binding isotherms obtained for the PDADMAC-P(St-co-SSNa) complexation. (**a**) Corrected heat rates obtained for the titration of PDADMAC (6 mM in monomers) into P(St-co-SSNa). The concentration of P(St-co-SSNa was 1 mM in monomers (charged and uncharged units), which explains the apparent lower binding stoichiometry before converting concentrations to Z); (**b**) binding isotherms corresponding to the various titration experiments as function of Z. The solid line represents the best fit curve derived from a two independent binding site model.

Isothermal titration calorimetry (ITC) was used to determine the thermodynamic parameters related to the complexation process between P(St-co-SSNa) and PDADMAC. Here, the P(St-co-SSNa) solutions were titrated by ITC with the PDADMAC solution, as in the case of DLS and zetametry experiments. The binding isotherms have the typical shape of a high affinity binding system, where ITC peaks are exothermic and of almost constant intensity throughout the titration, except near the equivalency where endothermic signals of low intensity can be detected (Figure 9). The data could not be satisfactorily fitted with a single set of identical site equation (data not shown). Much better data fitting was obtained by using a two independent binding site equation that has already been successfully applied to model the coacervation of two interacting macromolecules [43]. This model put forward that two thermodynamic processes take place during complexation, one exothermic and the other endothermic, both being almost constant in intensity until charge equivalency (Figure S5). This is consistent with a two-step complexation: the dominant exothermic contribution arises from the formation of primary complexes and the endothermic contribution from the formation of secondary complexes, as shown in previous ITC studies [60,61]. The primary complexation allows for a strong decrease in the electrostatic energy of the system by charge pairing between P(St-co-SSNa) and PDADMAC [62]. Concerning the secondary process, it is difficult to conclude on its endothermic nature since several attractive interactions may be responsible for the aggregation of primary complexes of similar charge (hydrophobic interactions, counterion correlations, charge fluctuations, charge nonuniformity, non-equilibrium hydrodynamic effects, and structural solvation interactions), all of which are summarized in [15]. A striking point is that the two processes are quite indistinguishable on the ITC peaks, suggesting that complexation II takes place concomitantly with complexation I (Figure 9a and Figure S5). In a similar work on PDADMAC/PSSNa complexation, Konko et al. could distinctly observe the presence of a shoulder after each injection peak; thus showing that secondary complexation follows primary complexation [63]. The reason for these discrepancies may be due to differences in PE molar masses, or concentrations or stirring speed in ITC. Here, endothermic peaks could only be distinctively detected near the charge equivalency, i.e., close to Z = 1, where the aggregation of complexes is maximal (Figure 9a). 

The thermodynamic parameters resulting from the fits are summarized in Table 2. For primary complexation, the enthalpy change (ΔH) associated with the ion pairing is largely dominated by the entropy contribution (TΔS) for all P(St-co-SSNa) systems investigated. This entropy gain, which can be attributed to the release of counterions and bound water molecules is known to be the driving force behind PEC formation [62]. One can also observe that the complexation enthalpy continuously decreases (in absolute value) when the degree of sulfonation of P(St-co-SSNa) decreased. The same holds for the free enthalpy change (ΔG) and complexation constant (K). Since the energy of an ion pair is a priori constant, this decrease in enthalpy should then result from an additional energy barrier required to reach and complex the anionic charges embedded in the hydrophobic domains (pearls or aggregates) of the partly sulfonated P(St-co-SSNa) chains. In all cases, the complexation must be total because the stoichiometry of the reaction is close to n = 1 for all compositions of P(St-co-SSNa) (Table 2). In other words, the hydrophobic domains in the P(St-co-SSNa) chains are not dense and cohesive enough to prevent PDADMAC from accessing and complexing the sulfonate groups. A feature that could have been anticipated from the AFM images (Figure 4) where only a few collapsed chains are present on the cationic mica even for the more hydrophobic PSS45%. This suggest that under strong complexation conditions, confirmed here by relatively high binding constants K (~10^6^ M^−1^), PE chains can adopt an open conformation favoring the juxtaposition of long portions of oppositely charged PEs, which is necessary to achieve the PDADMAC:P(St-co-SSNa) complexation with a stoichiometry near 1:1 [64].

For the secondary process (clustering of complexes I), the binding constants were one order of magnitude lower than for primary complexation. As for the primary complexation, the enthalpy term decreased when the degree of sulfonation decreased, suggesting that the two stages of complexation were closely related. Furthermore, since the endothermic contributions were almost constant throughout the titration up to the end point (Figure S5, black lines), the stoichiometry values of the secondary complexation were also close to 1, suggesting the concomitance of the primary and secondary complexation. 

### 3.5. Spectrophotometric Titration of P(St-co-SSNa) with Toluidine Blue

**Figure 10:**
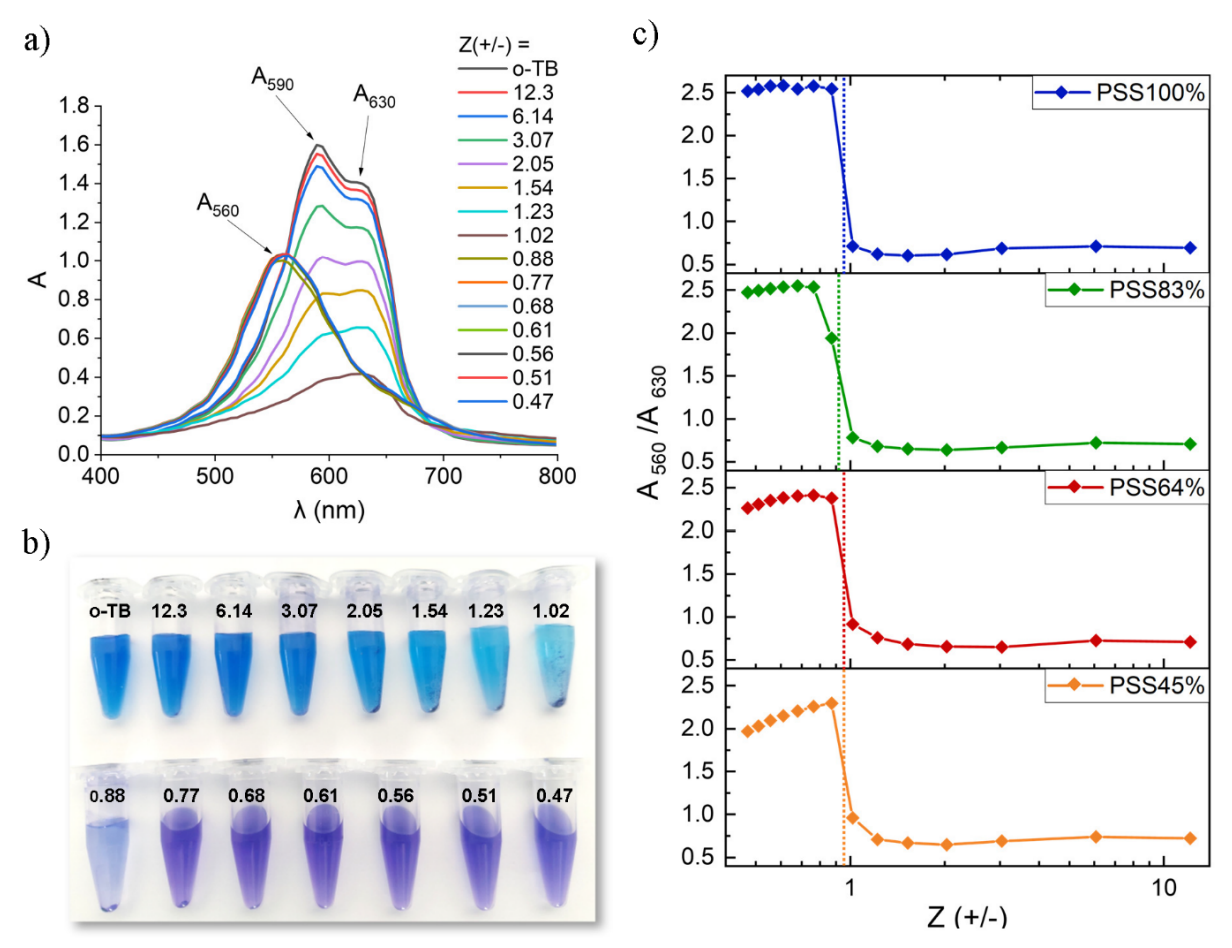
Spectrophotometric titration of P(St-co-SSNa) with toluidine blue (o-TB). (**a**) Absorbance spectra for PSS100% at various Z (+/−) ratios where the concentration in positive charges corresponds to the o-TB concentration. Three specific wavelengths are considered: 630 nm (monomeric o-TB), 590 nm (dimeric o-TB), and 560 nm (metachromatic peak); (**b**) aspect of the solutions at various Z ratios after centrifugation; (**c**) absorbance ratios (A_560_/A_630_) as function of Z(+/−) for the different P(St-co-SSNa) compositions. The charge equivalence is indicated by the dotted lines. Solid lines are only to guide eyes.

DLS, zetametry, and ITC all evidenced that complexation of PDADMAC and P(St-co-SSNa) is nearly stoichiometric in the sense of a 1:1 charge compensation with minimal differences between the different P(St-co-SSNa) compositions and then insensitive to the sulfonation rate. This unambiguously shows that the polyanion charges are available for complexation by a strong and hydrophilic PE, such as PDADMAC. However, what happens when P(St-co-SSNa) is complexed with a monovalent cationic molecule? To answer this question the complexation of P(St-co-SSNa) with a cationic dye, the o-toluidine blue (o-TB), was studied by spectrophotometry. o-TB is known to induce metachromacy upon binding with polyanions, as seen by a spectral shift towards shorter wavelengths (the color of solutions changes from blue to red-violet). The metachromacy of o-TB results from the stack of dye molecules bound to the polyanion favoring dye–dye interaction as in dye aggregates [65]. For example, o-TB is commonly used as an indicator for polyelectrolyte or colloidal titration [66,67]. P(St-co-SSNa) solutions varying in concentrations were incubated with a defined amount of o-TB. A color change was observed above a critical polymer concentration, which unambiguously showed that dyes could electrostatically bind to polyanions and aggregate; thus inducing metachromacy (Figure 10a,b). The stoichiometry of the complexation could then be accurately determined by plotting the ratio of the absorbance of the metachromatic peak (λ = 560 nm) to the free monomeric o-TB peak (λ = 630 nm) as a function of Z (+/−), with the concentration of cationic charges corresponding now to the o-TB concentration [68,69]. Figure 10c clearly shows that the binding stoichiometry of o-TB to P(St-co-SSNa) is close to 1:1 regardless of the degree of sulfonation of the polyanion, as previously reported for poly (ammonium acrylate) [68] and alginate [69]. The rather hydrophobic nature of the dye could explain the complete binding with the negative charges of P(St-co-SSNa) in the hydrophobic domains, but Ben Mahmoud et al. have shown with a similar dye, methylene blue, that the primary interaction is electrostatic [70]. The influence of the hydrophobicity of the dye could be verified by using hydrophilic dyes, but these generally do not induce metachromacy. Compared to data obtained with poly (ammonium acrylate) [68] and alginate [69], a striking feature in Figure 10c is that the change in the A_560_/A_630_ ratio is sharp on both sides of Z = 1. For Z > 1 (excess of dye), the absorbance ratio is consistently low revealing the absence of metachromatic effect, as shown by the color of the solutions (Figure 10b). The o-TB peak absorbance also decreased with increasing the polymer content suggesting part of the dye was removed from solution (Figure 10a). A careful examination of the solutions showed the formation of a violet precipitate, which was even more apparent after centrifugation of the solutions (Figure 10b). This clearly demonstrates that complexes were not stable in the presence of an excess of the monocationic dye. This is likely due to a lack of complex overcharging, a macromolecular feature well illustrated by stable primary complexes formed with PDADMAC, as well as the hydrophobic nature of P(St-co-SSNa) polyanions. The titration of excess o-TB in supernatants after centrifugation of complexes confirmed the binding stoichiometry close to 1:1 (Figure S6). In contrast, for Z < 1 (polymer in excess), there was almost no precipitate after centrifugation, suggesting the formation of stable and small metachromatic complexes. DLS measurements performed at Z < 1 confirmed the presence of well-defined colloidal structures with mean sizes below 100 nm, except for Z = 0.9 where some aggregation was detected (Figure S7). Zeta potential measurements indicated that the complexes are strongly negatively charged (Figure S7). In addition, both the sizes and the scattered intensities of complexes were similar before and after centrifugation, which suggests that complexes have a rather loose structure. The formation of hydrophobic o-TB stacks along the polyanion chains is expected to drive the intrapolymer association of P(St-co-SSNa) into unimolecular flower-like micelles, as observed for some amphiphilic copolymers bearing pendant hydrophobic blocks [71,72]. Another interesting feature of the system is that the A_560_/A_630_ ratio remains almost constant when the polymer concentration increases (i.e., Z tends to 0) indicating that dye molecules hardly distribute into smaller stacks in the presence of an excess of P(St-co-SSNa) (Figure 10c). This is consistent with the cooperative self-binding tendency of the dye, i.e., o-TB ions will favor a free binding site on the polyanion chain adjacent to a binding site that is already occupied by another o-TB ion (L.F.W. Vleugels, personal communication, 29 April 2022). In other words, the electrostatic interaction between o-TB and P(St-co-SSNa) is the driving force for binding, but o-TB stacking is the guiding theme for where o-TB ions are going to bind on the polyanion chain. The cooperative self-binding, however, is challenged by the decrease in charge density of the polyanions, as evidenced by the continuous decrease of A_560_/A_630_ for Z < 1 when the degree of sulfonation is reduced (Figure 10c and Figure S8). A similar trend was observed by using methylene blue instead of toluidine blue [70].

## 4. Conclusions

In this work, we studied the complexation of a hydrophobic polyanion (P(St-co-SSNa)) of variable charge density with a hydrophilic polycation (PDADMAC) using a set of complementary techniques ranging from TEM, DLS, AFM to ITC and spectrophotometric titration. Stable PEC particles with high (absolute) zeta potential were formed on both sides of the charge neutrality (Z < 1 or Z > 1). On the contrary, rather unstable PECs with low (absolute) zeta potential were formed close to charge stoichiometry (Z~1) leading to macroscopic precipitation with time. Furthermore, the results confirmed the existence of a two-step complexation mechanism. A first step where ion pairs are generated between oppositely charged PEs due to attractive electrostatic interactions inducing the formation of primary complexes. A second step where these primary PECs aggregate into secondary larger particles of finite size. More importantly, our data support that complexation of PDADMAC and P(St-co-SSNa) is nearly stoichiometric and insensitive to the sulfonation rate. On a thermodynamic point of view, ITC analyses showed that PE complexation (first step) was, as expected, largely entropy driven with a much larger entropic contribution than binding enthalpy, in line with the release of counterions and bound water molecules upon complexation. In all cases, the ion pairing step has always been characterized by exothermic enthalpies whereas aggregation (second step) by endothermic ones. In addition, the P(St-co-SSNa) charge density had a direct and measurable impact on the thermodynamic parameters computed in this study. The binding enthalpy, binding constant, and Gibbs free energy increased (in absolute value) with the charge density for the first complexation step. Finally, our data suggest that under strong complexation conditions highlighted here by relatively high binding constants (K ~ 10^6^ M^−1^), all anionic charges, embedded or not in the hydrophobic domains (pearls) of P(St-co-SSNa), are accessible and can be cooperatively complexed in the presence of the strong and hydrophilic cationic PDADMAC. When P(St-co-SSNa) was complexed with a monovalent cationic dye (o-toluidine blue), the complexation stoichiometry was also close to 1:1 at the point of charge equivalence, which demonstrates that the cooperative effect due to the macromolecular nature of PDADMAC is not essential to fully complex the charges of P(St-co-SSNa). Clearly, the electrostatic interaction with the strong (poly)cation overcomes the hydrophobic effect responsible for the pearl formation in P(St-co-SSNa). As a perspective to this work, it would be interesting to further investigate the role of various parameters on the clustering of primary complexes into finite-size aggregates, including not only the hydrophobicity of PEs, but also the ionic strength, PE concentrations, as well as the mixing conditions.

## Figures and Tables

**Table 1:** Characteristics of the PEs investigated in this work: charge, weight-average molecular weight (M_w_), degree of polymerization (N), chemical fraction of sulfonated units (*f*), charge spacing based on the monomer size (0.25 nm) (b_1_), mean nearest-neighbor distance of sulfonate groups based on the radial distribution function of sulfur atoms of P(St-co-SSNa) (b_2_).

Polymer	Charge	M_w_ (kDa)	N	*f* (%)	b_1_ (nm)	b_2_ (nm) ^b^	Name
PDADMAC	+	≤100	619	100	0.47 ^a^		PDADMAC
P(St-co-SSNa)	−	400	2692	45	0.56	0.80	PSS45%
P(St-co-SSNa)	−	450	2692	64	0.39	0.78	PSS64%
P(St-co-SSNa)	−	500	2692	83	0.30	0.77	PSS83%
P(St-co-SSNa)	−	550	2692	100	0.25	0.58	PSS100%

^a^ according to [38]; ^b^ see Figure S2.

**Table 2:** Thermodynamic parameters of the primary and secondary process obtained for the complexation of P(St-co-SSNa) by PDADMAC at 25 °C. n, K, ΔG, ΔH, and TΔS correspond to the reaction stoichiometry, the binding constant, the free energy change, the enthalpy change, and the entropy contribution to the free energy. The mole refers to the monomer unit of PDADMAC (titrant).

**Primary Process**	**n**	**K (M^−1^)**	**ΔG (kJ mol^−1^)**	**ΔH (kJ mol^−1^)**	**T** **ΔS (kJ mol^−1^)**
PSS100%	1.06	1.70.10^6^	−35.56	−8.66	26.90
PSS83%	1.10	1.39.10^6^	−35.06	−8.02	27.04
PSS64%	1.01	1.21.10^6^	−34.73	−7.50	27.23
PSS45%	1.02	7.39.10^5^	−33.50	−6.04	27.46
**Secondary Process**	**n**	**K (M^−1^)**	**ΔG (kJ mol^−1^)**	**ΔH (kJ mol^−1^)**	**T** **ΔS (kJ mol^−1^)**
PSS100%	1.08	3.23.10^5^	−31.44	7.00	38.44
PSS83%	1.13	2.87.10^5^	−31.14	5.94	37.08
PSS64%	1.07	5.55.10^5^	−32.79	5.60	38.39
PSS45%	1.07	5.68.10^5^	−32.85	5.32	38.17

## Data Availability

Not applicable.

## Supplementary Materials

The following supporting information can be downloaded at: https://www.mdpi.com/article/10.3390/polym14122404/s1, Table S1: Sulfonation degrees of the different P(St-co-SSNa) obtained by elemental analysis of carbon and sodium; Table S2: Refractive index increments (dn/dc) of P(St-co-SSNa); Figure S1: ^1^H NMR spectra of the four P(St-co-SSNa) copolymers; Figure S2: Radial distribution function of sulfur atoms of P(St-co-SSNa); Figure S3: Intensity-averaged size distributions of PDADMAC-P(St-co-SSNa) complexes; Figure S4: ITC dilution experiment of PDADMAC in water; Figure S5: ITC data fitted with a two independent binding site model; Figure S6: Determination of the binding stoichiometry of *o*-TB with P(St-co-SSNa); Figure S7: Intensity-averaged size distributions of colloidal complexes obtained from PSS100% and *o*-toluidine blue in presence of an excess of polyanion; Figure S8: Variation of the metachromacy as function of the sulfonation rate for two Z ratios below 1 (polyanion in excess). Refs. [43,69,73] cited in Supplementary Materials.

## References

[1] Meka V.S., Sing M.K.G., Pichika M.R., Nali S.R., Kolapalli V.R.M., Kesharwani P. (2017). A comprehensive review on polyelectrolyte complexes. Drug Discov. Today.

[2] Thunemann A.F., Müller M., Dautzenberg H., Joanny J.F.O., Lowne H. (2004). Polyelectrolyte Complexes. Adv. Polym. Sci..

[3] Kabanov V., Decher G., Schlenoff J.B. (2003). Fundamentals of Polyelectrolyte Complexes in Solution and the Bulk. Multilayer Thin Films.

[4] Tsuchida E., Abe K. (1982). Interactions between macromolecules in solution and intermacromolecular complexes. Adv. Polym. Sci..

[5] Philipp B., Dautzenberg H., Linow K.J., Koetz J., Dawydoff W. (1989). Polyelectrolyte complexes—recent developments and open problems. Prog. Polym. Sci..

[6] Nasser M.S., Twaiq F.A., Onaizi S.A. (2013). Effect of Polyelectrolytes on the Degree of Flocculation of Papermaking Suspensions. Sep. Purif. Technol..

[7] Schmitt C., Turgeon S.L. (2011). Protein/polysaccharide Complexes and Coacervates in Food Systems. Adv. Colloid Interface Sci..

[8] Crini G. (2005). Recent Developments in Polysaccharide-Based Materials Used as Adsorbents in Wastewater Treatment. Prog. Polym. Sci..

[9] Kulkarni A.D., Vanjari Y.H., Sancheti K.H., Patel H.M., Belgamwar V.S., Surana S.J., Pardeshi C.V. (2016). Polyelectrolyte complexes: Mechanisms, critical experimental aspects, and applications. Artif. Cells Nanomed. Biotechnol..

[10] Li H., Fauquignon M., Haddou M., Schatz C., Chapel J.-P. (2021). Interfacial Behavior of Solid- and Liquid-like Polyelectrolyte Complexes as a Function of Charge Stoichiometry. Polymers.

[11] Wang Q., Schlenoff J.B. (2014). The Polyelectrolyte Complex/Coacervate Continuum. Macromolecules.

[12] Dautzenberg H. (1997). Polyelectrolyte complex formation in highly aggregating systems. 1. Effect of salt: Polyelectrolyte complex formation in the presence of NaCl. Macromolecules.

[13] Kabanov V.A., Zezin A.B. (1984). A new class of complex water-soluble polyelectrolytes. Makromol. Chem..

[14] Dautzenberg H., Hartmann J., Grunewald S., Brand F. (1996). Stoichiometry and structure of polyelectrolyte complex particles in diluted solutions. Ber. Bunsenges. Phys. Chem..

[15] Lebovka N.I., Müller M. (2014). Aggregation of Charged Colloidal Particles. Polyelectrolyte Complexes in the Dispersed and Solid State I: Principles and Theory.

[16] Starchenko V., Müller M., Lebovka N.I. (2012). Sizing of PDADMAC/PSS Complex Aggregates by Polyelectrolyte and Salt Concentration and PSS Molecular Weight. J. Phys. Chem..

[17] Brand F., Dautzenberg H. (1997). Structural Analysis in Interpolyelectrolyte Complex Formation of Sodium Poly (styrenesulfonate) and Diallyldimethylammonium Chloride–Acrylamide Copolymers by Viscometry. Langmuir.

[18] Mende M., Schwarz S., Zschoche S., Petzold G., Janke A. (2011). Influence of the Hydrophobicity of Polyelectrolytes on Polyelectrolyte Complex Formation and Complex Particle Structure and Shape. Polymers.

[19] Gummel J., Cousin F., Boué B. (2008). Structure Transition in PSS/Lysozyme Complexes: A Chain-Conformation-Driven Process, as Directly Seen by Small Angle Neutron Scattering. Macromolecules.

[20] Gummel J., Boué B., Clemens D., Cousin F. (2008). Finite size and inner structure controlled by electrostatic screening in globular complexes of proteins and polyelectrolyte. Soft Matter.

[21] Dautzenberg H., Görnitz E., Jaeger W. (1998). Synthesis and characterization of poly (diallyldimethylammonium chloride) in a broad range of molecular weight. Macromol. Chem. Phys..

[22] Ben Mahmoud S., Essafi W., Brûlet A., Boué F. (2018). How necklace pearls evolve in hydrophobic polyelectrolyte chains under good solvent addition: A SANS study of the conformation. Macromolecules.

[23] Dautzenberg H., Rother G.J. (1988). Interpretation of light scattering from supermolecular structures in liquid systems by master curves. J. Polym. Sci. Polym. Phys..

[24] Dautzenberg H., Rother G. (1992). Supermolecular structures in polymer solutions interpretation of static light scattering data. Makromol. Chem. Macromol. Symp..

[25] Dobrynin A.V., Rubinstein M., Obukhov S.P. (1996). Cascade of Transitions of Polyelectrolytes in Poor Solvents. Macromolecules.

[26] Dobrynin A.V., Rubinstein M. (1999). Hydrophobic polyelectrolytes. Macromolecules.

[27] Baigl D., Sferrazza M., Williams C.E. (2003). On the pearl size of hydrophobic polyelectrolytes. Europhys. Lett..

[28] Spiteri M.N., Williams C.E., Boué F. (2007). Pearl-Necklace-Like Chain Conformation of Hydrophobic Polyelectrolyte: A SANS Study of Partially Sulfonated Polystyrene in Water. Macromolecules.

[29] Essafi W., Lafuma F., Williams C.E. (1995). Effect of Solvent Quality on the Behaviour of Highly Charged Polyelectrolytes. J. Phys..

[30] Carbajal-Tinocco M.D., Williams C.E. (2000). Static properties of hydrophobic polyelectrolytes in the thermodynamic limit. Europhys. Lett..

[31] Manning G.S. (1969). Limiting Laws and Counterion Condensation in Polyelectrolyte Solutions I. Colligative Properties. J. Chem. Phys..

[32] Oosawa F. (1971). Polyelectrolytes.

[33] Essafi W., Lafuma F., Baigl D., Williams C.E. (2005). Anomalous counterion condensation in salt-free hydrophobic polyelectrolyte solutions: Osmotic pressure measurements. Europhys. Lett..

[34] Gromer A., Rawiso M., Maaloum M. (2008). Visualization of Hydrophobic Polyelectrolytes Using Atomic Force Microscopy in Solution. Langmuir.

[35] Essafi W., Lafuma F., Williams C.E., Schmitz K.S. (1994). Structure of polyelectrolyte solutions at intermediate charge densities. Macro-ion Characterization. From Dilute Solutions to Complex Fluids.

[36] Makowski H.S., Lundberg R.D., Singhal G.S. (1975). Flexible Polymeric Compositions Comprising a Normally Plastic Polymer Sulfonated to about 0.2 to about 10 Mole % Sulfonate. U.S. Patent.

[37] Baigl D., Seery T.A.P., Williams C.E. (2002). Preparation and Characterization of Hydrosoluble, Partially Charged Poly(styrenesulfonate)s of Various Controlled Charge Fractions and Chain Lengths. Macromolecules.

[38] Lorchat P., Konko I., Combet J., Jestin J., Johner A., Laschewski A., Obukhov S., Rawiso M. (2014). New regime in polyelectrolyte solutions. EPL (Europhys. Lett.).

[39] Shen X.-C., Bao L., Zhang Z.-L., Liu X., Pang D.-W., Xu J. (2011). A simple and effective sample preparation method for atomic force microscopy visualization of individual DNA molecules in situ. Mol. Biol. Rep..

[40] Pastré D., Hamon L., Landousy F., Sorel I., David M.-O., Zozime A., Le Cam E., Piétrement O. (2006). Anionic Polyelectrolyte Adsorption on Mica Mediated by Multivalent Cations: A Solution to DNA Imaging by Atomic Force Microscopy under High Ionic Strengths. Langmuir.

[41] Wiseman T., Williston S., Brandts J.F., Lin L.N. (1989). Rapid measurement of binding constants and heats of binding using a new titration calorimeter. Anal. Biochem..

[42] Pierce M.M., Raman C.S., Nall B.T. (1999). Isothermal titration calorimetry of protein-protein interactions. Methods.

[43] Aberkane L., Jasniewski J., Gaiani C., Scher J., Sanchez C. (2010). Thermodynamic Characterization of Acacia Gum-β-Lactoglobulin Complex Coacervation. Langmuir.

[44] Brautigam C.A. (2015). Fitting two- and three-site binding models to isothermal titration calorimetric data. Methods.

[45] Colby R.H. (2010). Structure and linear viscoelasticity of flexible polymer solutions: Comparison of polyelectrolyte and neutral polymer solutions. Rheol. Acta.

[46] Sedlák M. (1999). What Can Be Seen by Static and Dynamic Light Scattering in Polyelectrolyte Solutions and Mixtures?. Langmuir.

[47] Muthukumar M. (2016). Ordinary–extraordinary transition in dynamics of solutions of charged macromolecules. Proc. Natl. Acad. Sci. USA.

[48] Sedlák M. (1997). Dynamic light scattering from binary mixtures of polyelectrolytes. II. Appearance of the medium polyelectrolyte mode upon mixing and comparison with experiments on binary mixtures of neutral polymers. J. Chem. Phys..

[49] Balding P., Borrelli R., Volkovinsky R., Russo P.S. (2022). Physical Properties of Sodium Poly(styrene sulfonate): Comparison to Incompletely Sulfonated Polystyrene. Macromolecules.

[50] Balding P., Cueto R., Russo P.S., Gutekunst W.R. (2019). Synthesis of perfectly sulfonated sodium polystyrene sulfonate over a wide molar mass range via reversible-deactivation radical polymerization. J. Polym. Sci. Polym. Chem..

[51] Stradner A., Sedgwick H., Cardinaux F., Poon W.C., Egelhaaf S.U., Schurtenberger P. (2004). Equilibrium cluster formation in concentrated protein solutions and colloids. Nature.

[52] Pandav G., Pryamitsyn V., Ganesan V. (2015). Interactions and Aggregation of Charged Nanoparticles in Uncharged Polymer Solutions. Langmuir.

[53] Starchenko V., Müller M., Lebovka N. (2008). Growth of polyelectrolyte complex nanoparticles: Computer simulations and experiments. J. Phys. Chem..

[54] Qi L., Fresnais J., Berret J.-F., Castaing J.-C., Grillo I., Chapel J.-P. (2010). Influence of the Formulation Process in Electrostatic Assembly of Nanoparticles and Macromolecules in Aqueous Solution: The Mixing Pathway. J. Phys. Chem..

[55] Qi L., Fresnais J., Berret J.-F., Chapel J.-P. (2010). Influence of the formulation process in electrostatic assembly of nanoparticles and macromolecules in aqueous solution: The interaction pathway. J. Phys. Chem..

[56] Dautzenberg H., Radeva T. (2001). Polyelectrolyte complex formation in highly aggregating systems: Methodical aspects and general tendencies. Physical Chemistry of Polyelectrolytes.

[57] Yuana Y., Gaoa J., Zhaia Y., Li D., Fu C., Huanga Y. (2022). Mixing efficiency affects the morphology and compactness of chitosan/tripolyphosphate nanoparticles. Carbohydr. Polym..

[58] Ryde N. (2014). Re: Do Polymer Molecules Have Zeta Potential?. https://www.researchgate.net/post/Do_polymer_molecules_have_zeta_potential/53665293d3df3e80648b456d/citation/download.

[59] Oshima H., Delgado A.V. (2002). Electrophoresis of Charged Particles and Drops. Interfacial Electrokinetics and Electrophoresis.

[60] Vitorazi L., Ould-Moussa N., Sekar S., Fresnais J., Loh W., Chapel J.-P., Berret J.-F. (2014). Evidence of a two-step process and pathway dependency in the thermodynamics of poly(diallyldimethylammonium chloride)/poly(sodium acrylate) complexation. Soft Matter.

[61] Delas T., Mock-Joubert M., Faivre J., Hofmaier M., Sandre O., Dole F., Chapel J.-P., Crépet A., Trombotto S., Delair T. (2019). Effects of Chain Length of Chitosan Oligosaccharides on Solution Properties and Complexation with siRNA. Polymers.

[62] Ou Z., Muthukumar M. (2006). Entropy and enthalpy of polyelectrolyte complexation: Langevin dynamics Simulations. J. Chem. Phys..

[63] Konko I. (2015). Aqueous Solutions of Complexes Formed by Model Polyelectrolytes of Opposite Charges. Doctoral Dissertation.

[64] Michaels A.S., Mir L., Schneider N.S. (1965). A Conductometric Study of Polycation—Polyanion Reactions in Dilute Aqueous Solution. J. Phys. Chem..

[65] Pal M.K., Chaudhuri M. (1970). Conductometric titrations of anionic polyelectrolytes with metachromatic dyes and effects of organic solvents. Die Makromol. Chem. Macromol. Chem. Phys..

[66] Terayama H. (1952). Method of colloid titration (a new titration between polymer ions). J. Polym. Sci..

[67] Horn D., Heuck C.C. (1983). Charge determination of proteins with polyelectrolyte titration. J. Biol. Chem..

[68] Ben Fradj A., Lafi R., Gzara L., Hamzaoui A.H., Hafiane A. (2014). Spectrophotometric study of the interaction of toluidine blue with poly (ammonium acrylate). J. Mol. Liq..

[69] Vleugels L.F.W., Ricois S., Voets I.K., Tuinier R. (2017). Reversal of metachromasy revisited; displacement of Toluidine-blue from alginate by surfactants. Colloids Surf. Physicochem. Eng. Asp..

[70] Ben Mahmoud S., Hamzaoui A.H., Essafi W. (2016). Spectrophotometric study of the interaction of methylene blue with poly (styrene-co-sodium styrene sulfonate). Mediterr. J. Chem..

[71] Yusa S.-I. (2012). Self-Assembly of Cholesterol-Containing Water-Soluble Polymers. Int. J. Polym. Sci..

[72] Lee K.Y., Jo W.H., Kwon I.C., Kim Y.-H., Jeong S.Y. (1998). Structural Determination and Interior Polarity of Self-Aggregates Prepared from Deoxycholic Acid-Modified Chitosan in Water. Macromolecules.

[73] Baigl D. (2003). Etude Expérimentale de Polyélectrolytes Hydrophobes Modèles. Ph.D. Thesis.

