# Supplementary material for: Complexation in Aqueous Solution of a Hydrophobic Polyanion (PSSNa) Bearing Different Charge Densities with a Hydrophilic Polycation (PDADMAC)
Source: Polymers (Basel). 2022 Jun 14;14(12):2404. doi: 10.3390/polym14122404 (PMC9229680; doi:10.3390/polym14122404)
Supplement: Supplementary file 1 [file polymers-14-02404-s001.zip › polymers-1748899-supplementary.pdf]

# Complexation in aqueous solution of a hydrophobic polyanion (PSSNa) bearing different charge densities with a hydrophilic polycation (PDADMAC)

Nouha Jemili [1,2], Martin Fauquignon [3], Etienne Grau [3], Nicolas Fatin-Rouge [4], François Dole [5], Jean-Paul Chapel [5], Wafa Essafi [1,2], Christophe Schatz [3]

[1] Laboratoire Matériaux, Traitement et Analyse, Institut National de Recherche et d'Analyse Physico-Chimique, Pôle Technologique de Sidi Thabet, 2020 Sidi Thabet, Tunisie
[2] Université de Tunis El Manar, Faculté des Sciences de Tunis, 2092 Tunis, Tunisie
[3] Laboratoire de Chimie des Polymères Organiques (LCPO), Université de Bordeaux, CNRS, Bordeaux INP, UMR 5629, 33600 Pessac, France
[4] Institut de Chimie des Milieux et des Matériaux de Poitiers, Université de Poitiers, 86073 Poitiers, France
[5] Centre de Recherche Paul Pascal (CRPP), UMR CNRS 5031, Université de Bordeaux, 33600 Pessac, France

**Table S1.** Sulfonation degrees of the different P(St-co-SSNa) obtained by elemental analysis of carbon and sodium: $f\ (\%) = 100 \times \frac{96 \times w_{Na}}{23 \times w_C}$ with $w_{Na}$ and $w_c$ the mass fractions of sodium and carbon respectively.

| Polymer | % Carbon | % Sodium | Sulfonation degree f (%) |
|---|---|---|---|
| PSS45% | 58.5 | 6.3 | 44.7 |
| PSS64% | 51.0 | 7.8 | 64.2 |
| PSS83% | 44.8 | 8.9 | 83.0 |
| PSS100% | 39.4 | 9.4 | 99.4 |

**Table S2.** Refractive index increments (dn/dc) of P(St-co-SSNa) at $\lambda$=658 nm

| P(St-co-SSNa) | dn/dc (mL/g)[a] |
|---|---|
| PSS100% | $0.1617 \pm 0.1021$ |
| PSS83% | $0.1669 \pm 0.1283$ |
| PSS64% | $0.1840 \pm 0.0960$ |
| PSS45% | $0.1859 \pm 0.1198$ |

[a] determined with an Optilab reX refractive index detector (Wyatt, Santa Barbara, US) from a range of five P(St-co-SSNa) concentrations prepared in 0.01 M NaOH

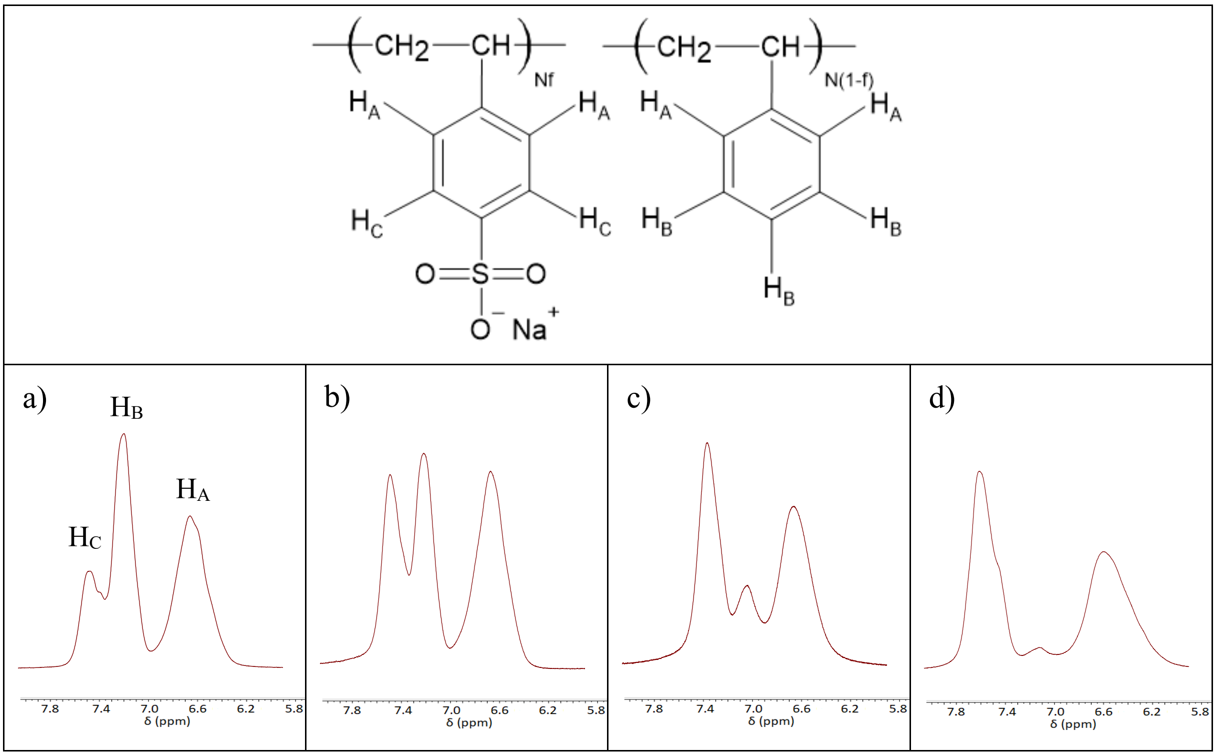


**Figure S1.** $^{1}$H NMR spectra of the four P(St-co-SSNa) copolymers: a) PSS45% in d-DMSO, b) PSS64% in d-DMSO, c) PSS83% in $D_2O$ and d) PSS100% in $D_2O$. The sulfonation degrees (*f*) were obtained according to the equation: $f\ (\%) = 100\ \times \frac{3\alpha_{C/B}}{2 + 3\alpha_{C/B}}$ with $\alpha_{C/B}$ the ratio of the heights of peaks C and B [1]. The following values of $f$ were found: 42.2%, 62.4%, 81.5% and 95% for PSS45%, PSS64%, PSS83% and PSS100% respectively.

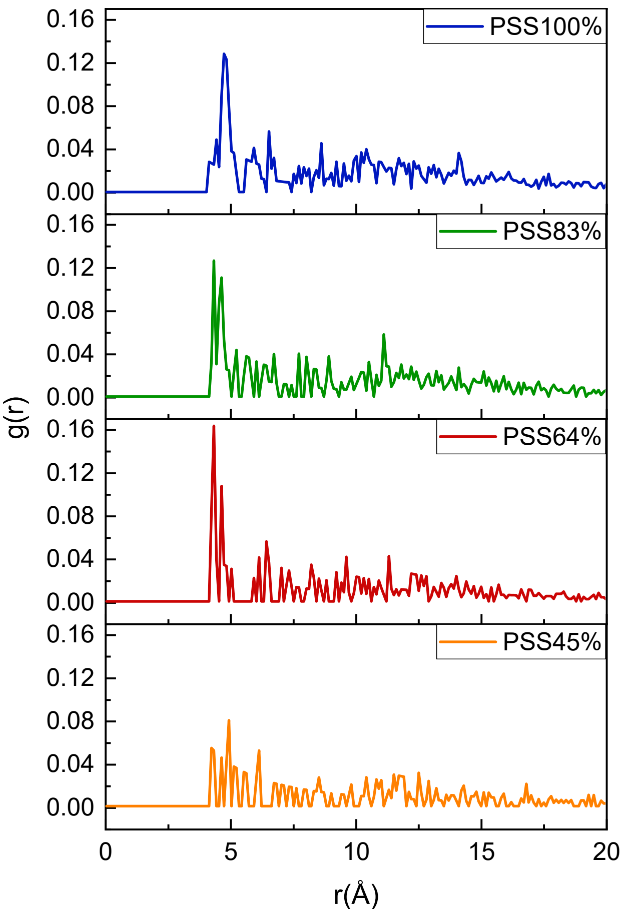


**Figure S2.** Radial distribution functions for sulfur atoms of P(St-co-SSNa) (N=100 units) around themselves. The area under the curve within a given distance interval is proportional to the probability of finding a sulfur atom.

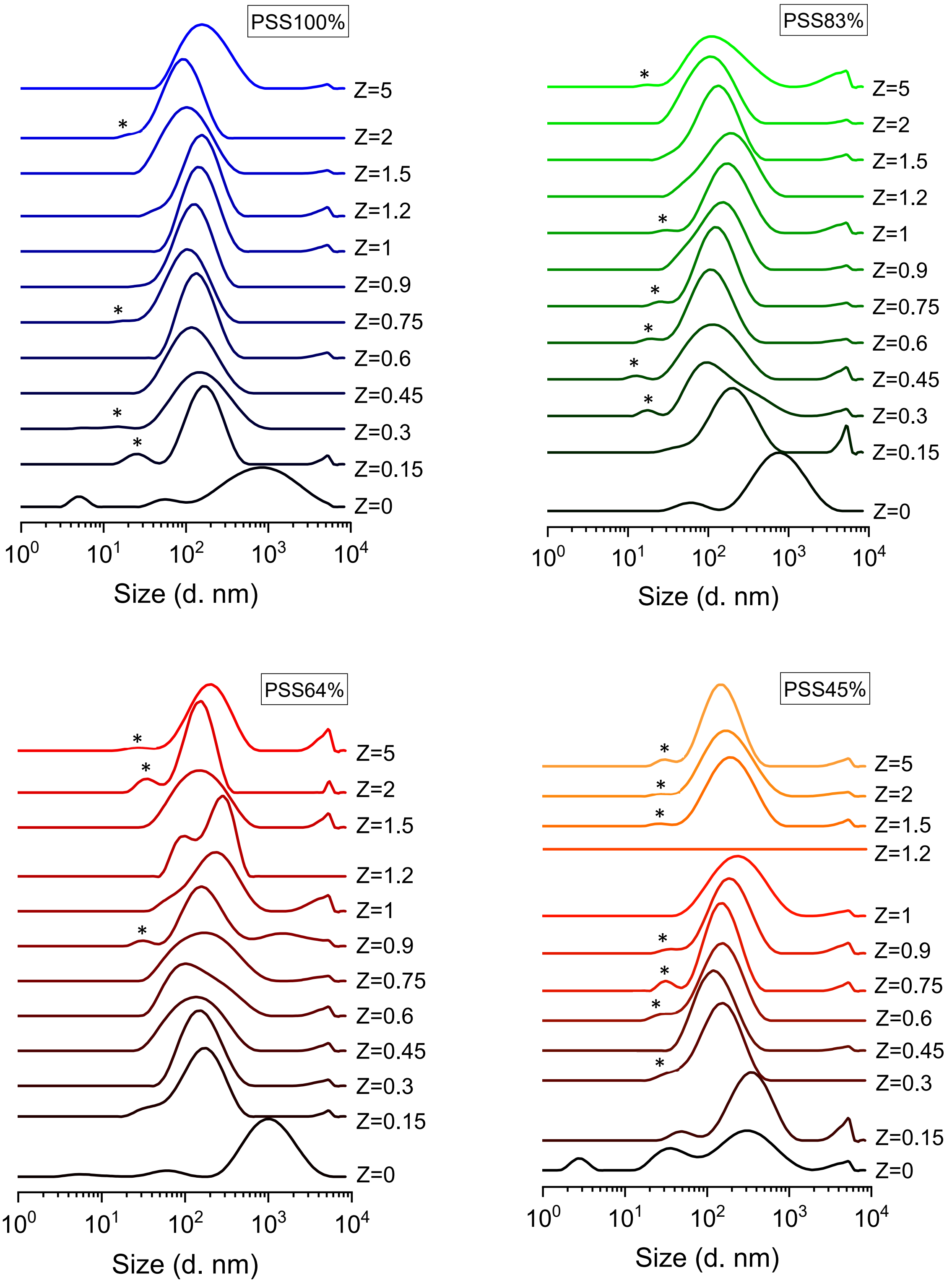


**Figure S3.** Intensity-averaged size distributions of PDADMAC-P(St-co-SSNa) complexes prepared at different charge ratios Z(+/-) by rapid addition of PDADMAC to P(St-co-SSNa) for Z < 1 (or P(St-co-SSNa) to PDADMAC for Z >1). The total concentration in PE was equal to 1.5 mM. The asterisks indicate the possible presence of primary complexes coexisting with secondary complexes (main population). Due to their low contribution to the overall scattering intensity, they were not systematically detected for all Z ratios. A flat size distribution was obtained for PSS45% at Z=1.2 due to the full precipitation of complexes.

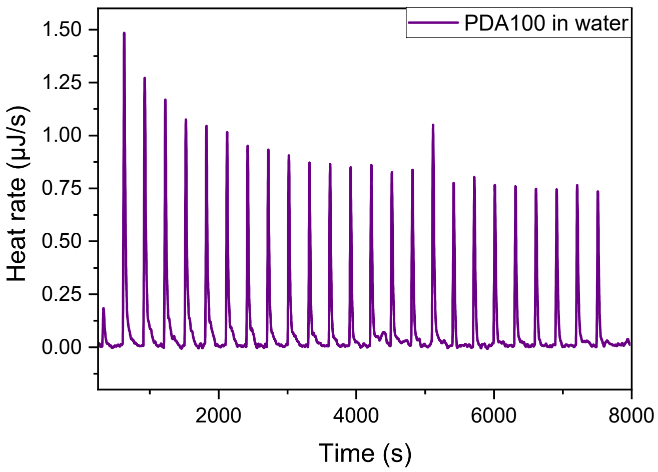


**Figure S4.** ITC dilution experiment of PDADMAC at 6 mM (in monomer units) in water.

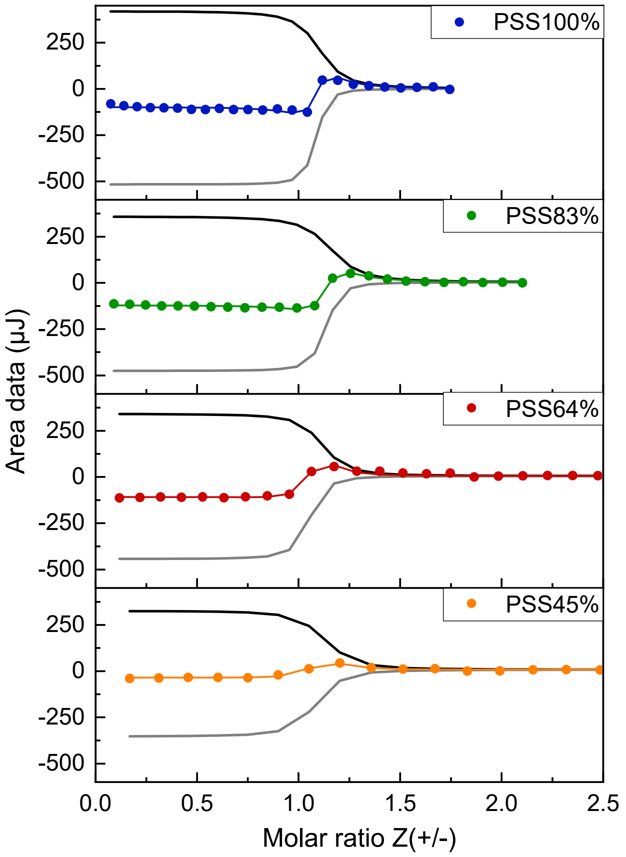


**Figure S5.** ITC data fitted with a two-independent binding site model [2], plotted as a function of the molar charge ratio Z(+/-) for the titration of PSS45%, PSS64%, PSS83% and PSS100% by PDADMAC. The grey and black lines represent the ion-pairing (exothermic) and the aggregation (endothermic) contributions to the overall process, respectively. The continuous coloured lines represent the overall fits to the experimental data.

**Determination of the binding stoichiometry of *o*-TB with P(St-co-SSNa)**

In addition to the method reported in Figure 10.c, the binding stoichiometry of *o*-TB with P(St-co-SSNa) was also determined from the titration of excess *o*-TB in the supernatants of P(St-co-SSNa)/*o*-TB mixtures after centrifugation. 1.35 mL of *o*-TB solution prepared at $1.2.10^{-4}$ M was rapidly added to 0.15 mL of P(St-co-SSNa) solutions varying in concentration, from $5.10^{-5}$ M to $1.2.10^{-3}$M (in charged residues). The solutions were incubated at 25°C under stirring for one hour and then centrifuged (20 min, 10,000 g) to allow the insoluble complex particles to sediment. Triplicates of supernatant were pipetted into a 96 well plate (200 µL/well) and analysed with a microplate reader (SpectraMax M2e, Molecular Devices) in the visible range (400-800 nm). The *o*-TB concentration was determined from a calibration curve plotted at 590 nm (dimeric *o*-TB) which was found to be linear in a larger range of concentrations than at 630 nm (monomeric *o*-TB) [3].

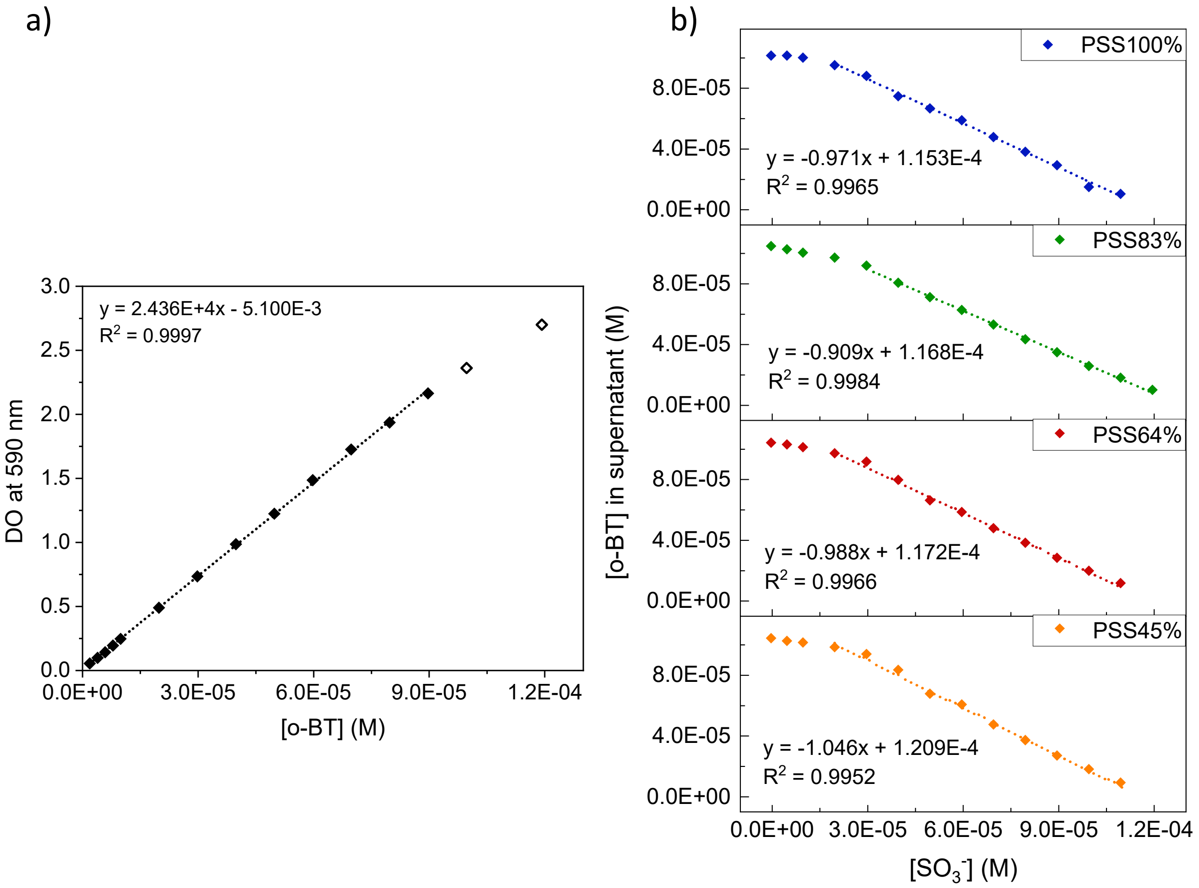


**Figure S6**. Determination of the binding stoichiometry of *o*-TB with P(St-co-SSNa). a) Calibration Curve relating the optical density (OD) measured at 590 nm (dimeric dye) with the concentration of *o*-TB. The linearity is observed up to a concentration of $9.10^{-5}$ M in the conditions used here. b) Variation of the concentration of excess o-TB (after centrifugation) as function of the P(St-co-SSNa) concentration, expressed in sulfonate groups. The binding stoichiometry was obtained from the absolute value of the slope of the linear regression at 590 nm. Note that the deviation to Lambert-Beer's law and/or the formation of complexes that could not be sedimented by centrifugation causes downward deviation from the linearity at low polymer concentrations. These data points were not considered in the linear regression.

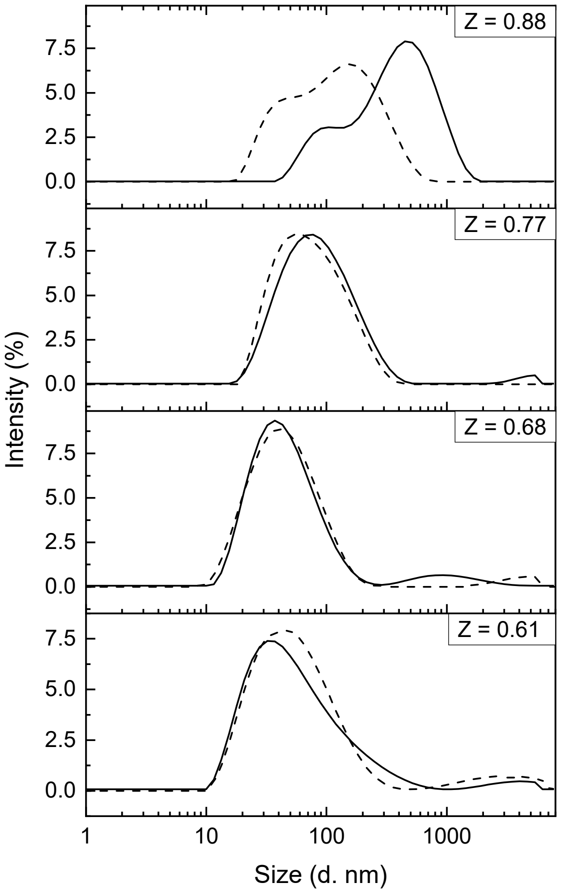


| Z | Derived count rate (kcps) Before centrifugation | Derived count rate (kcps) After centrifugation | Zeta potential (mV) |
|---|---|---|---|
| **0.88** | 28430 | 12430 | -33.3 ± 1.0 |
| **0.77** | 12950 | 12000 | -51.1 ± 0.3 |
| **0.68** | 2600 | 2670 | -48.3 ± 1.6 |
| **0.61** | 2390 | 2420 | -45.8 ± 1.1 |

**Figure S7**. Intensity-average diameter distributions of complexes obtained from PSS100% and *o*-toluidine blue in presence of an excess of polyanion at Z=0.88, Z=0.77, Z=0.68 and Z=0.61. All complex dispersions are violet and look homogeneous (no sedimentation). The size distributions are plotted before (plain lines) and after (dashed lines) centrifugation of the dispersions (10 min, 15,000 g). The scattering intensities are given as the derived count rates determined at 173° detection angle. Zeta potential values are also indicated.

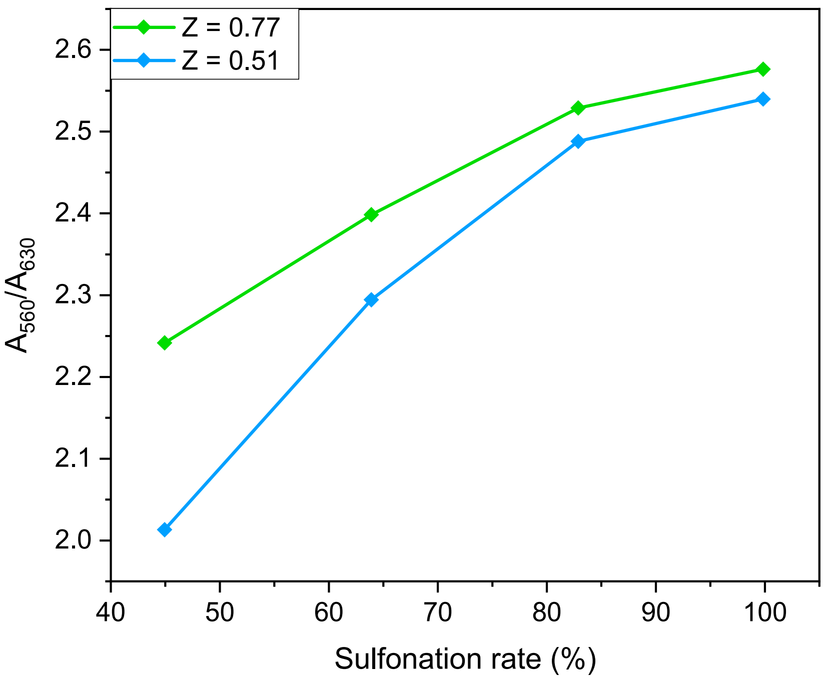


**Figure S8**. Variation of the metachromacy as function of the sulfonation rate for two Z ratios below 1 (polyanion in excess).

## References

[1] Baigl, D. (2003) Etude expérimentale de polyélectrolytes hydrophobes modèles ; PhD Thesis, Université Pierre et Marie Curie, Paris.
[2] Aberkane, L.; Jasniewski, J.; Gaiani, C.; Scher, J.; Sanchez C. Thermodynamic Characterization of Acacia Gum-β-Lactoglobulin Complex Coacervation. *Langmuir* **2010**, *26*, 12523–12533.
[3] Vleugels, L.F.W.; Ricois, S. Voets, I.K.; Tuinier, R. Reversal of metachromasy revisited; displacement of Toluidine-blue from alginate by surfactants. Colloids and Surfaces A: Physicochemical and Engineering Aspects, 2017, 529, 454–461.
